# Supplementary material for: Analyzing the worldwide progression of COVID-19 cases and deaths using nonlinear mixed-effects model
Source: PLoS One. 2024 Aug 12;19(8):e0306891. doi: 10.1371/journal.pone.0306891 (PMC11318863; doi:10.1371/journal.pone.0306891)
Supplement: S1 Fig — (PDF) [file pone.0306891.s001.pdf]

S1 Fig. Density plots of the estimated model parameters

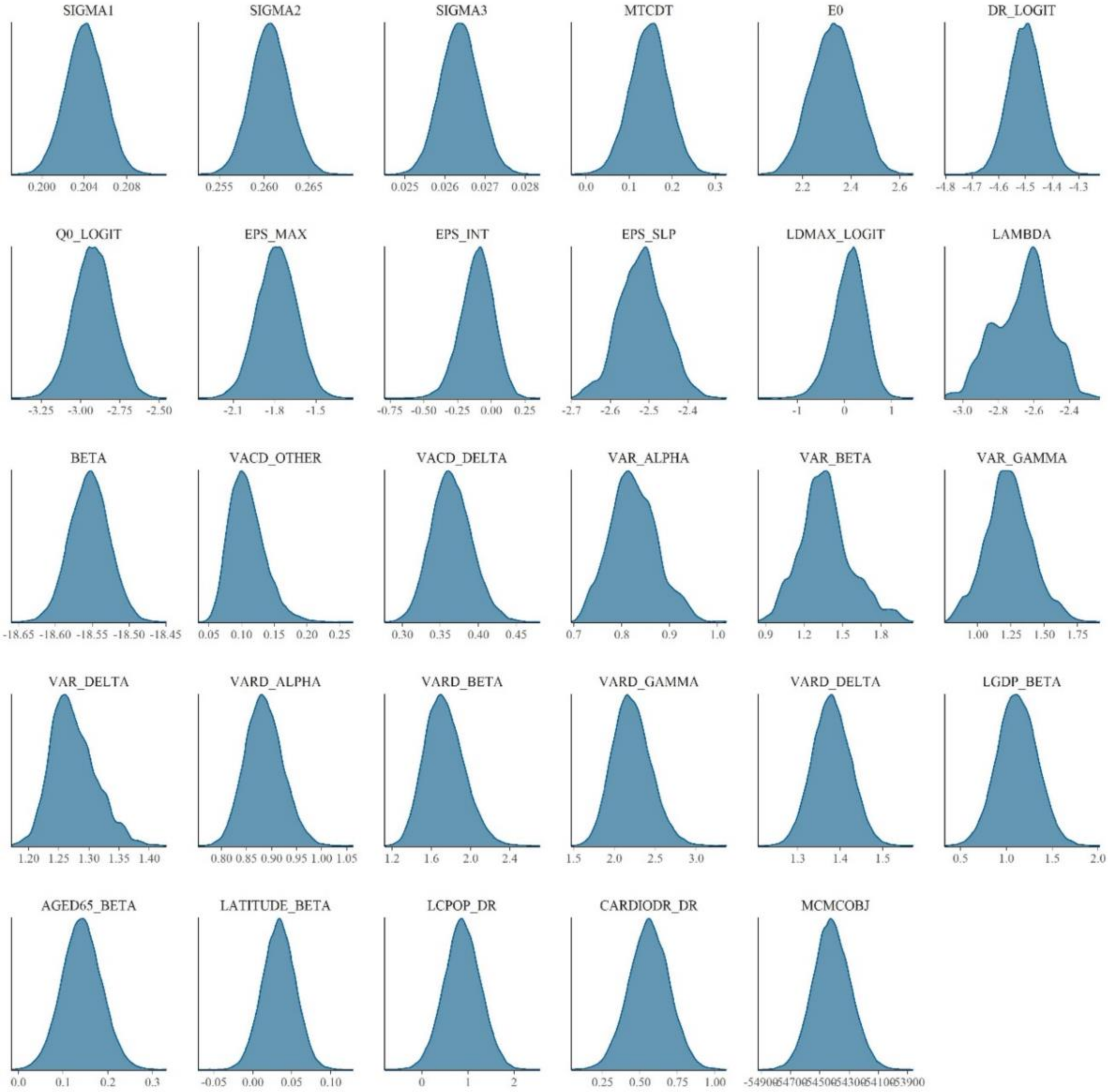

SIGMA1: proportional residual error of weekly confirmed cases (σ_1), SIGMA2: proportional residual error of weekly confirmed deaths (σ_2), SIGMA3: proportional residual error of weekly excess deaths (σ_3), MTCDT: mean time from infection to death (MT_{CDT}), E0: initial value of population in exposed compartment (E_0), DR_LOGIT: logit of death rate of COVID-19 in the infected patients for wild type virus variant (dr), Q0_LOGIT: logit of initial value of quarantined proportion in each compartment (Q_0), EPS_MAX: maximum rate of quarantine for symptomatic population (ε_{max}), EPS_INT: intercept of ε (ε_{int}), EPS_SLP: slope of ε (ε_{slp}), LDMAX_LOGIT: logit of maximum value of lockdown effect (LD_{max}), LAMBDA: rate of decrease of lockdown effect (λ), BETA: rate of transmission of infection for wild type virus variant (β), VACD_OTHER: odds ratio of vaccination effect on the death rate for virus variants other than delta compared to non-vaccinated population ($VacD_{Others}$), VACD_DELTA: odds ratio of vaccination effect on the death rate for delta virus variant compared to non-vaccinated population ($VacD_{Delta}$), VAR_ALPHA: odds ratio of transmissibility of infection for Alpha variant to wild type variant (Var_{Alpha}), VAR_BETA: odds ratio of transmissibility of infection for Beta variant to wild type variant (Var_{Beta}), VAR_GAMMA: odds ratio of transmissibility of infection for Gamma variant to wild

type variant (Var_{Gamma}), VAR_DELTA: odds ratio of transmissibility of infection for Delta variant to wild type variant (Var_{Delta}), VARD_ALPHA: odds ratio of death rate for Alpha variant to wild type variant ($VarD_{Alpha}$), VARD_BETA: odds ratio of death rate for Beta variant to wild type variant ($VarD_{Beta}$), VARD_GAMMA: odds ratio of death rate for Gamma variant to wild type variant ($VarD_{Gamma}$), VARD_DELTA: odds ratio of death rate for Delta variant to wild type variant ($VarD_{Delta}$), LGDP_BETA: effect of logarithmic of GDP on β, AGED65_BETA: effect of proportion of 65 years or older on β, LATITUDE_BETA: effect of latitude of capital city on β, LCPOP_DR: effect of logarithmic of city population on the death rate, CARDIODR_DR: Effect of cardiovascular death rate on the death rate, MCMCOBJ: objective function for Markov Chain Monte Carlo method.
