# Supplementary material for: Analyzing the worldwide progression of COVID-19 cases and deaths using nonlinear mixed-effects model
Source: PLoS One. 2024 Aug 12;19(8):e0306891. doi: 10.1371/journal.pone.0306891 (PMC11318863; doi:10.1371/journal.pone.0306891)
Supplement: S2 Fig — (PDF) [file pone.0306891.s002.pdf]

S2 Fig. Reported and predicted weekly confirmed cases in each country. Gray bar: reported weekly confirmed cases. Blue line: predicted weekly confirmed cases. The population in each country was normalized to 10^8 population in the analysis.

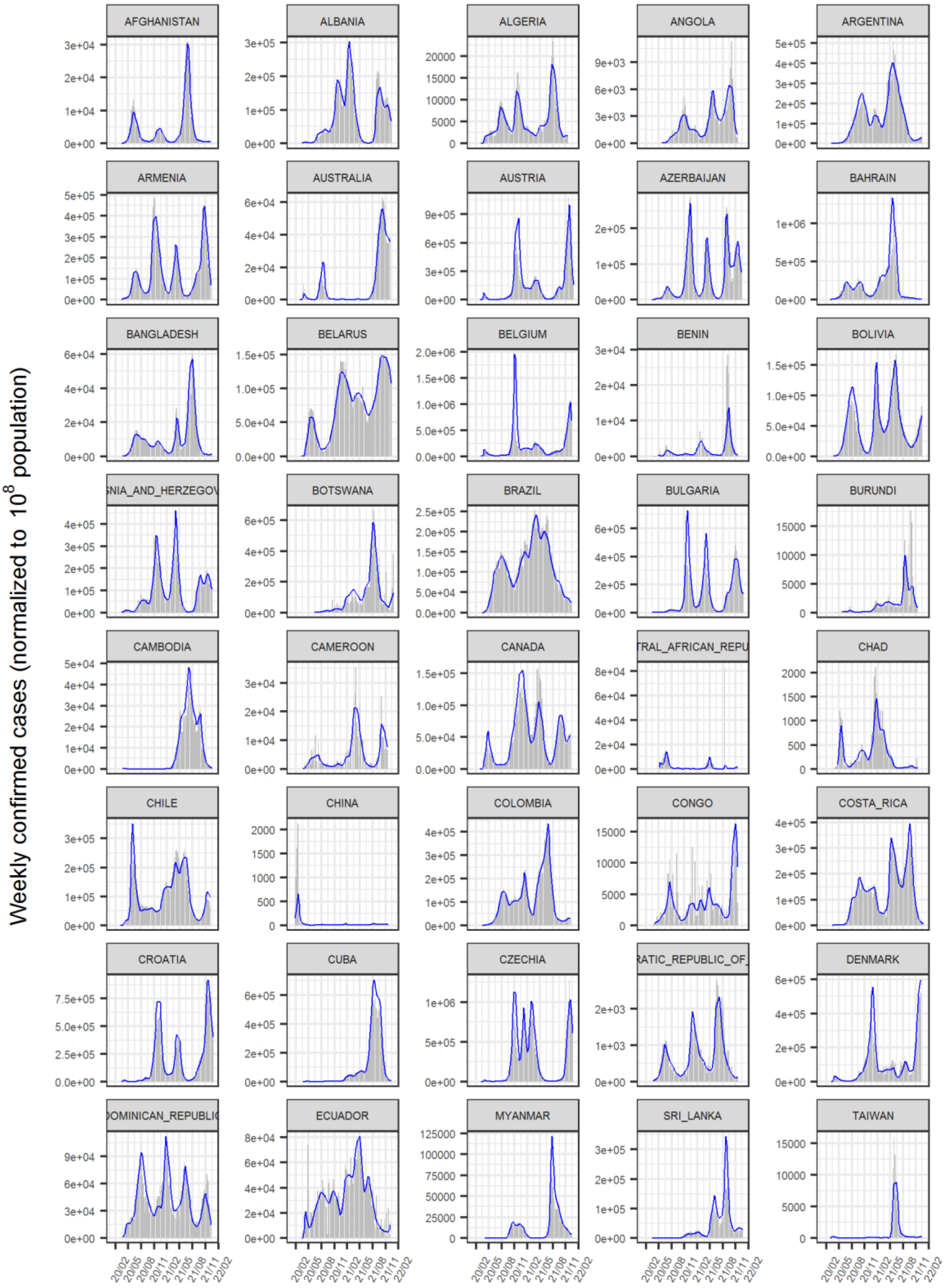

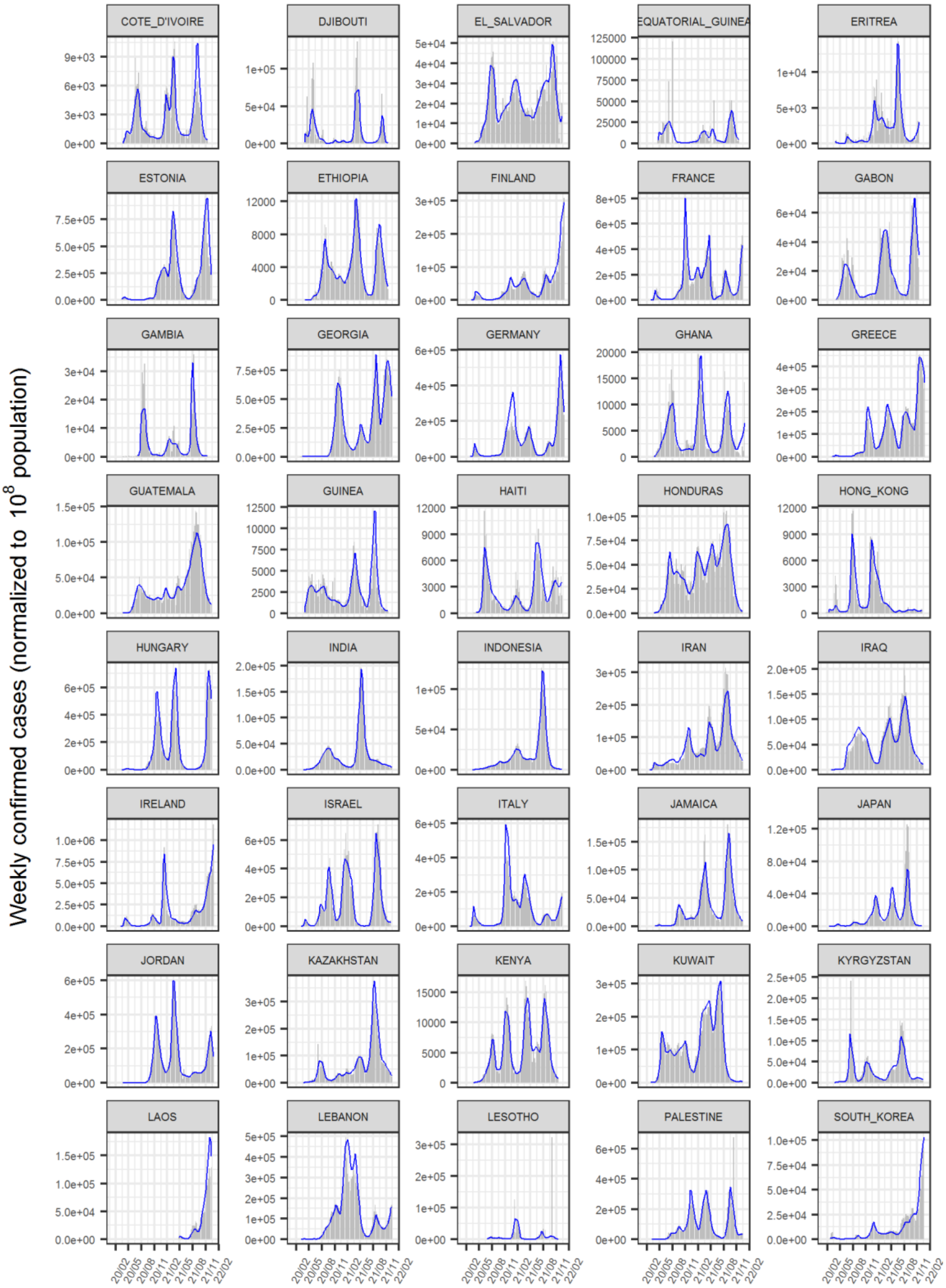
Weekly confirmed cases (normalized to 10^8 population)
COTE_D'IVOIRE
DJIBOUTI
EL_SALVADOR
EQUATORIAL_GUINEA
ERITREA
ESTONIA
ETHIOPIA
FINLAND
FRANCE
GABON
GAMBIA
GEORGIA
GERMANY
GHANA
GREECE
GUATEMALA
GUINEA
HAITI
HONDURAS
HONG_KONG
HUNGARY
INDIA
INDONESIA
IRAN
IRAQ
IRELAND
ISRAEL
ITALY
JAMAICA
JAPAN
JORDAN
KAZAKHSTAN
KENYA
KUWAIT
KYRGYZSTAN
LAOS
LEBANON
LESOTHO
PALESTINE
SOUTH_KOREA
20/02
20/05
20/08
20/11
21/02
21/05
21/08
21/11
22/02

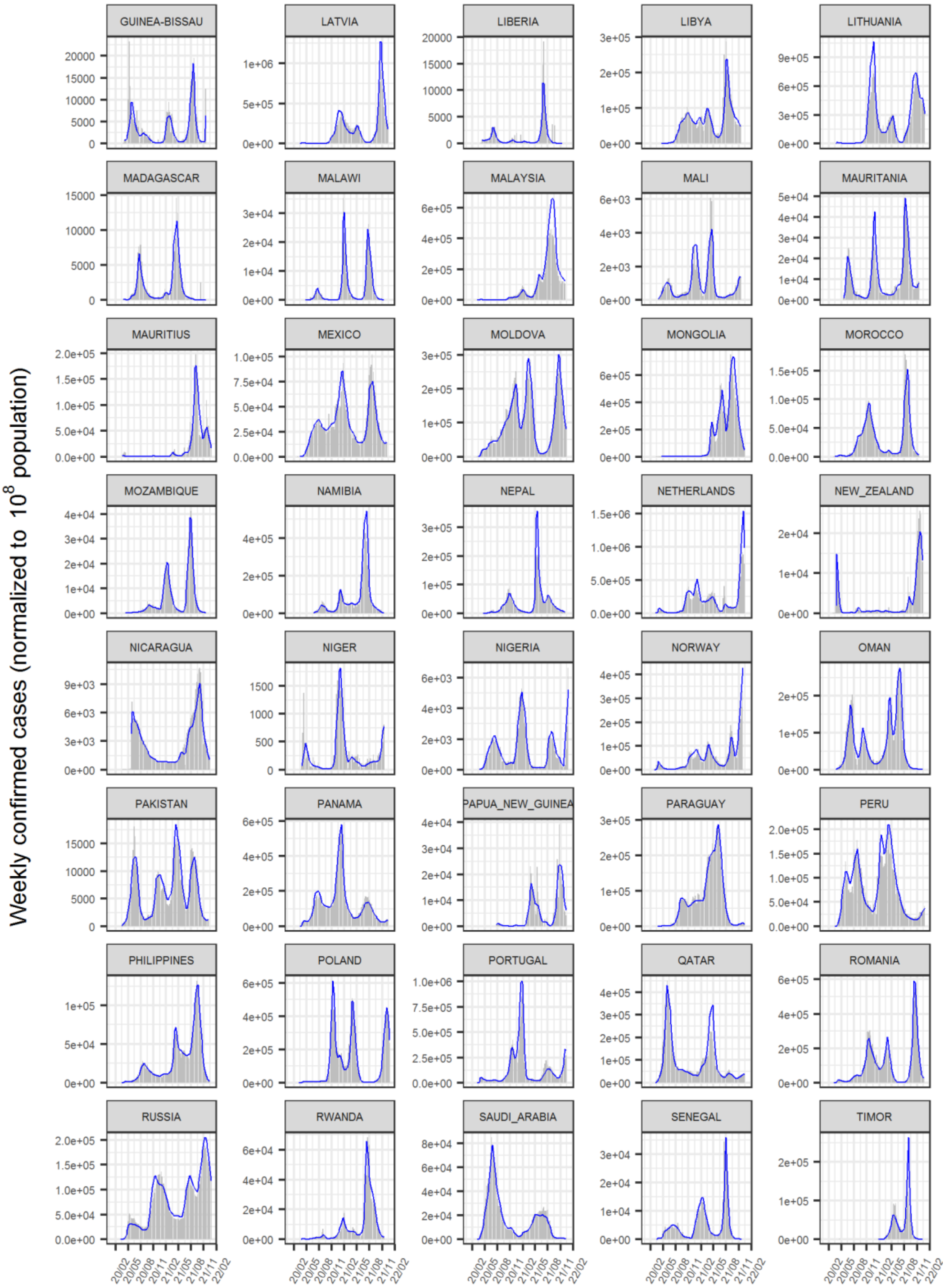
Weekly confirmed cases (normalized to 10^8 population)
GUINEA-BISSAU
LATVIA
LIBERIA
LIBYA
LITHUANIA
MADAGASCAR
MALAWI
MALAYSIA
MALI
MAURITANIA
MAURITIUS
MEXICO
MOLDOVA
MONGOLIA
MOROCCO
MOZAMBIQUE
NAMIBIA
NEPAL
NETHERLANDS
NEW_ZEALAND
NICARAGUA
NIGER
NIGERIA
NORWAY
OMAN
PAKISTAN
PANAMA
PAPUA_NEW_GUINEA
PARAGUAY
PERU
PHILIPPINES
POLAND
PORTUGAL
QATAR
ROMANIA
RUSSIA
RWANDA
SAUDI_ARABIA
SENEGAL
TIMOR
20/02 20/05 20/08 20/11 21/02 21/05 21/08 21/11 22/02

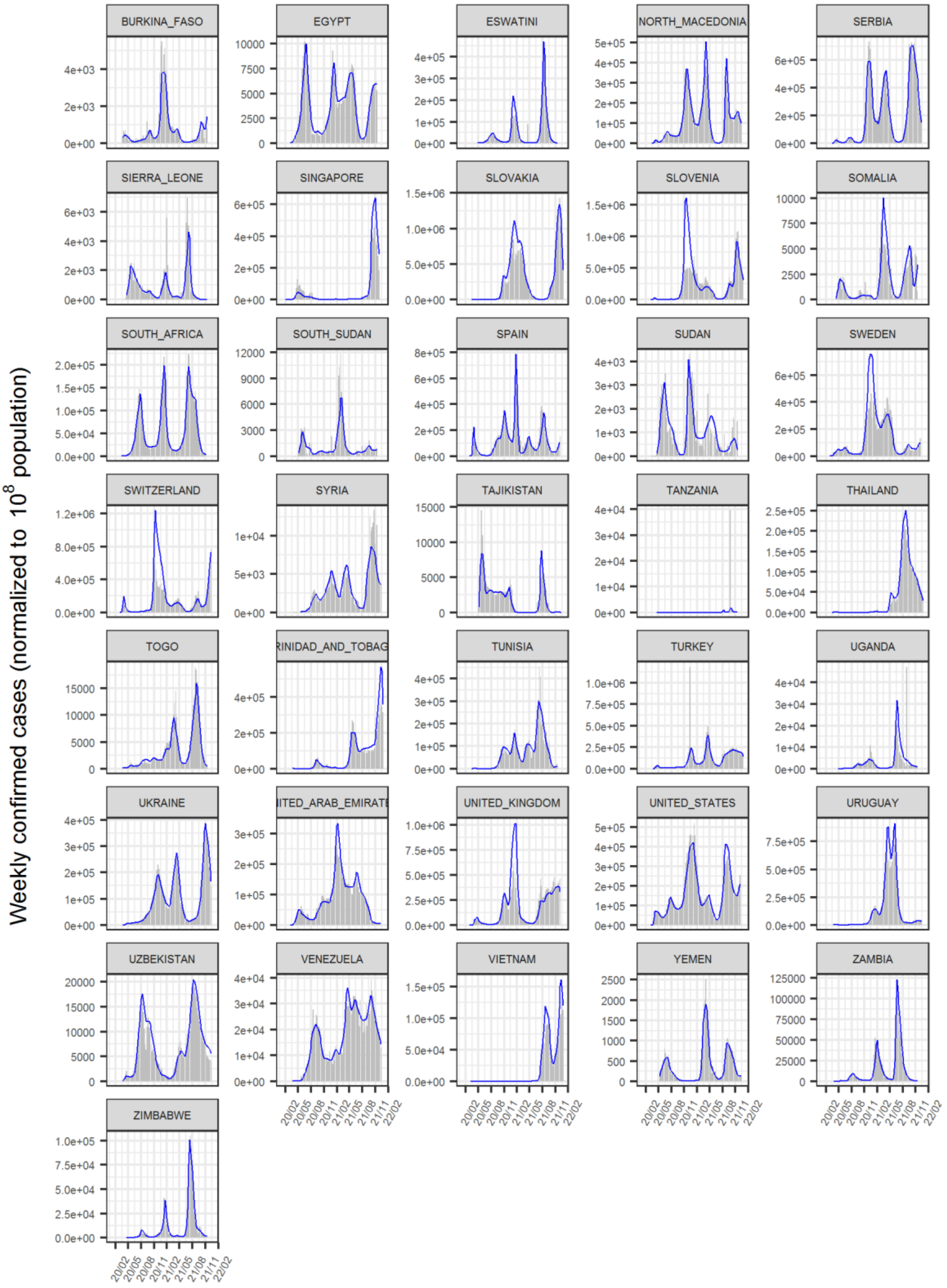
Weekly confirmed cases (normalized to 10^8 population)
BURKINA_FASO
EGYPT
ESWATINI
NORTH_MACEDONIA
SERBIA
SIERRA_LEONE
SINGAPORE
SLOVAKIA
SLOVENIA
SOMALIA
SOUTH_AFRICA
SOUTH_SUDAN
SPAIN
SUDAN
SWEDEN
SWITZERLAND
SYRIA
TAJIKISTAN
TANZANIA
THAILAND
TOGO
RINIDAD_AND_TOBAG
TUNISIA
TURKEY
UGANDA
UKRAINE
IITED_ARAB_EMIRATE
UNITED_KINGDOM
UNITED_STATES
URUGUAY
UZBEKISTAN
VENEZUELA
VIETNAM
YEMEN
ZAMBIA
ZIMBABWE
20/02
20/05
20/08
20/11
21/02
21/05
21/08
21/11
22/02
