# Supplementary material for: Analyzing the worldwide progression of COVID-19 cases and deaths using nonlinear mixed-effects model
Source: PLoS One. 2024 Aug 12;19(8):e0306891. doi: 10.1371/journal.pone.0306891 (PMC11318863; doi:10.1371/journal.pone.0306891)

S4 Fig. Reported and predicted weekly total deaths in each country. Total death is a sum of COVID-19 related confirmed death, COVID-19 related not confirmed death, and death from other reasons. Gray bar: reported weekly total deaths. Red line: predicted weekly total deaths. Green line and blue line: mean and its 90% confidence intervals of expected death from other reasons. The population in each country was normalized to 10^8 population in the analysis.

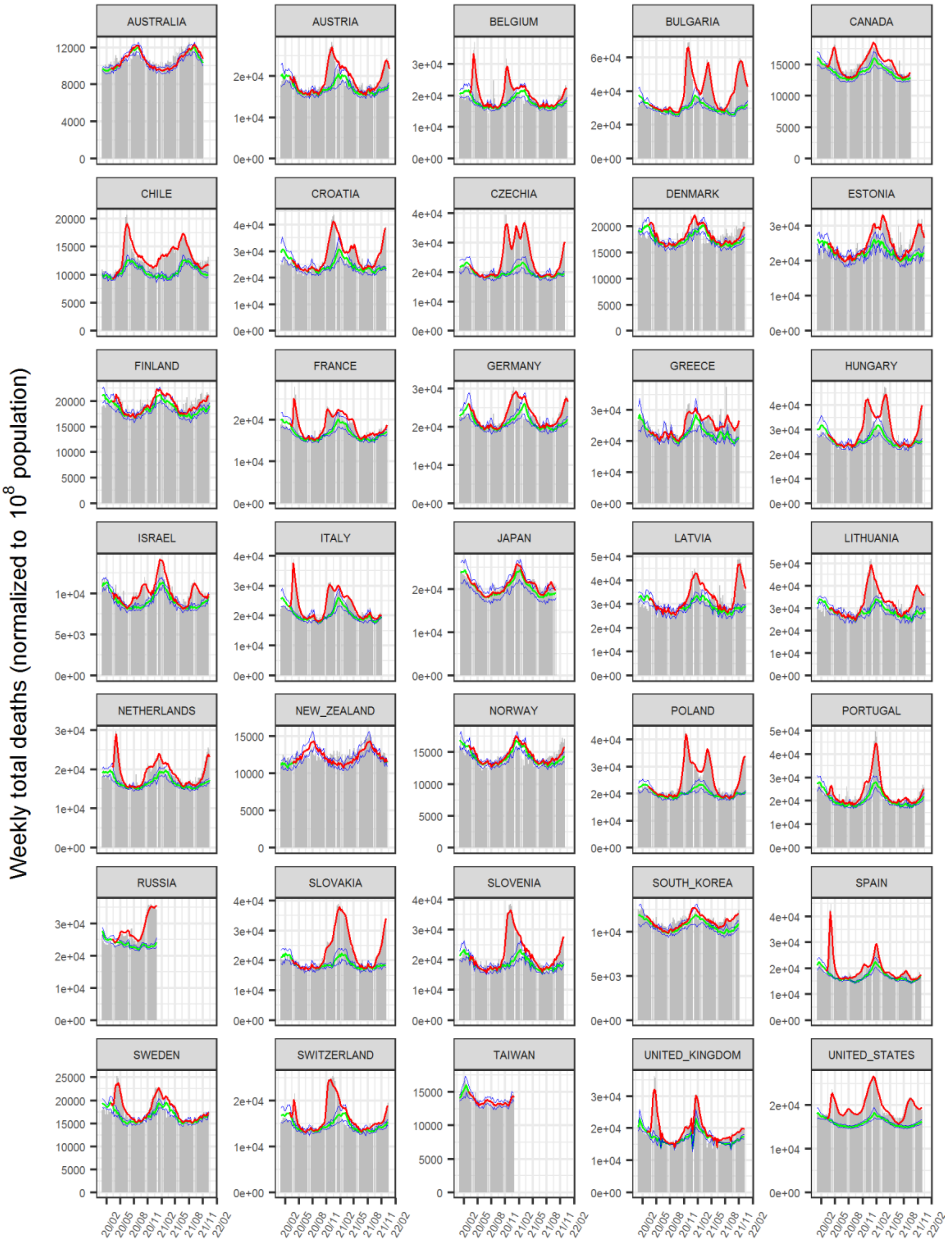

Supplement: S4 Fig — (PDF) [file pone.0306891.s004.pdf]
