# Supplementary material for: Analyzing the worldwide progression of COVID-19 cases and deaths using nonlinear mixed-effects model
Source: PLoS One. 2024 Aug 12;19(8):e0306891. doi: 10.1371/journal.pone.0306891 (PMC11318863; doi:10.1371/journal.pone.0306891)

S5 Fig. Simulated weekly cases and deaths if there were no vaccines available in the world.

(a) Simulated weekly cases of COVID-19 with and without vaccination

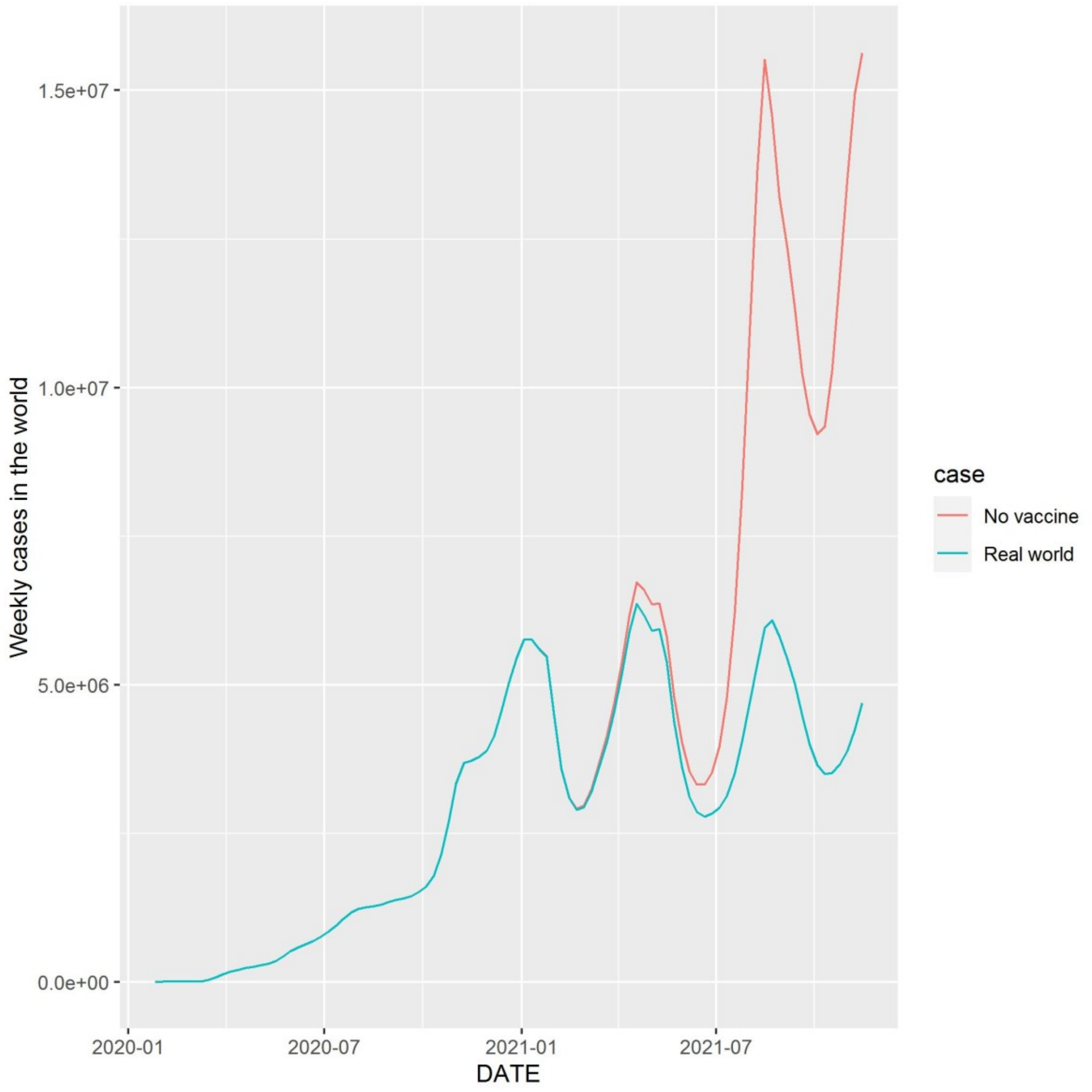

(b) Simulated weekly deaths by COVID-19 with and without vaccination

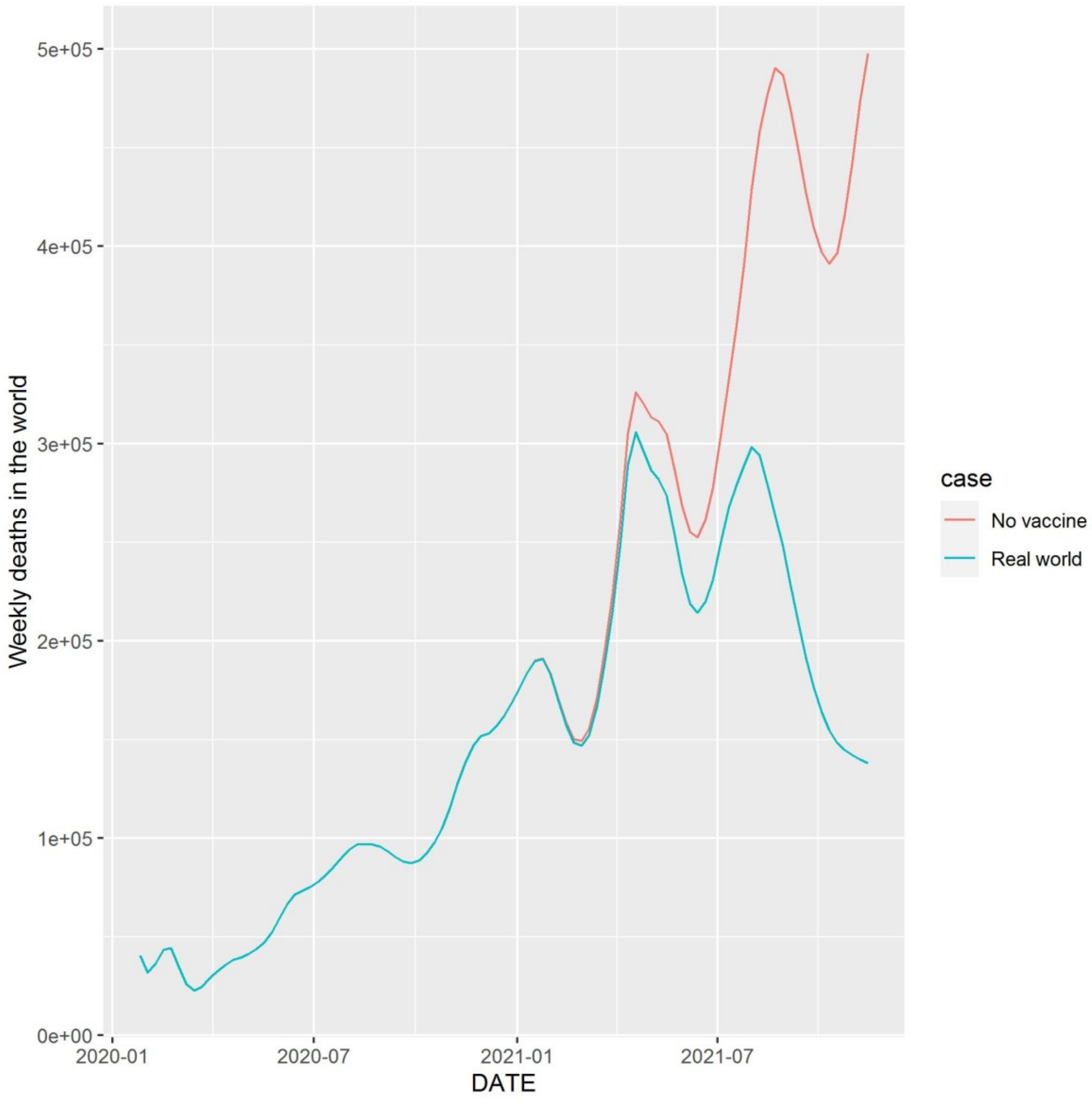

Supplement: S5 Fig — (PDF) [file pone.0306891.s005.pdf]
