# Supplementary material for: Analyzing the worldwide progression of COVID-19 cases and deaths using nonlinear mixed-effects model
Source: PLoS One. 2024 Aug 12;19(8):e0306891. doi: 10.1371/journal.pone.0306891 (PMC11318863; doi:10.1371/journal.pone.0306891)
Supplement: S1 Table — (DOCX) [file pone.0306891.s006.docx]

**S1 Table.** **Lockdown start and end date in each country, territory, and place.** Downloaded from Wikipedia as of 15 Jan 2022, modified for clarity. (https://en.wikipedia.org/wiki/COVID-19_lockdowns)

| **Country_territory** | **Place** | **Number of lockdown  in the country** | **Lockdown Start date** | **Lockdown End date** | **Lockdown_Length (days)** | **Level** |
| --- | --- | --- | --- | --- | --- | --- |
| Albania | Nationwide | 1st | 2020/3/13 | 2020/6/1 | 80 | National |
| Algeria | Algiers | 1st | 2020/3/23 | 2020/5/14 | 52 | City |
| Algeria | Blida | 1st | 2020/3/23 | 2020/5/14 | 52 | City |
| Argentina | Greater Buenos Aires | 1st | 2020/3/19 | 2020/11/8 | 234 | Metropolitan area |
| Argentina | Greater Buenos Aires | 2nd | 2021/5/22 | 2021/5/30 | 9 | Metropolitan area |
| Argentina | Greater Buenos Aires | 3rd | 2021/6/5 | 2021/6/6 | 2 | Metropolitan area |
| Argentina | Rest of the country | 1st | 2020/3/19 | 2020/5/10 | 52 | National |
| Argentina | Rest of the country | 2nd | 2021/5/22 | 2021/5/30 | 9 | National |
| Argentina | Rest of the country | 3rd | 2021/6/5 | 2021/6/6 | 2 | National |
| Armenia | Nationwide | 1st | 2020/3/24 | 2020/5/4 | 41 | National |
| Australia | Albury (NSW) | 2nd | 2021/8/14 | 2021/9/10 | 28 | Local government area |
| Australia | Albury (NSW) | 3rd | 2021/9/16 | 2021/9/23 | 7 | Local government area |
| Australia | Alice Springs | 2nd | 2021/6/30 | 2021/7/3 | 3 | Metropolitan area |
| Australia | Armidale | 2nd | 2021/8/7 | 2021/9/10 | 34 | Local government area |
| Australia | Australian Capital Territory | 2nd | 2021/8/12 | 2021/10/15 | 61 | Territory |
| Australia | Ballarat | 1st | 2020/3/31 | 2020/5/12 | 43 | Local government area |
| Australia | Ballarat | 2nd | 2020/8/6 | 2020/9/16 | 41 | Local government area |
| Australia | Ballarat | 3rd | 2021/2/13 | 2021/2/17 | 5 | Local government area |
| Australia | Ballarat | 4th | 2021/5/28 | 2021/6/3 | 7 | Local government area |
| Australia | Ballarat | 5th | 2021/7/16 | 2021/7/27 | 12 | Local government area |
| Australia | Ballarat | 6th | 2021/8/5 | 2021/8/9 | 5 | Local government area |
| Australia | Ballarat | 7th | 2021/8/21 | 2021/9/9 | 20 | Local government area |
| Australia | Ballarat | 8th | 2021/9/16 | 2021/9/22 | 7 | Local government area |
| Australia | Ballina | 2nd | 2021/8/9 | 2021/9/10 | 32 | Local government area |
| Australia | Bega Valley | 2nd | 2021/8/14 | 2021/9/16 | 33 | Local government area |
| Australia | Brisbane | 2nd | 2021/1/8 | 2021/1/11 | 3 | Metropolitan area |
| Australia | Brisbane | 3rd | 2021/3/29 | 2021/4/1 | 3 | Metropolitan area |
| Australia | Brisbane | 4th | 2021/6/29 | 2021/7/3 | 4 | Metropolitan area |
| Australia | Brisbane | 5th | 2021/7/31 | 2021/8/8 | 8 | Metropolitan area |
| Australia | Byron Shire | 2nd | 2021/8/9 | 2021/9/10 | 32 | Local government area |
| Australia | Byron Shire | 3rd | 2021/9/21 | 2021/9/28 | 7 | Local government area |
| Australia | Cairns Region | 2nd | 2021/8/8 | 2021/8/11 | 3 | Region |
| Australia | Casino | 2nd | 2021/8/14 | 2021/9/10 | 28 | Town |
| Australia | Casino | 3rd | 2021/10/2 | 2021/10/11 | 9 | Town |
| Australia | Central West | 2nd | 2021/7/20 | 2021/7/27 | 7 | Region |
| Australia | Central West | 3rd | 2021/8/14 | 2021/10/11 | 58 | Region |
| Australia | Cowra | 2nd | 2021/8/14 | 2021/9/10 | 28 | Local government area |
| Australia | Cowra | 3rd | 2021/9/20 | 2021/10/5 | 14 | Local government area |
| Australia | Darwin | 2nd | 2021/6/27 | 2021/7/2 | 5 | Metropolitan area |
| Australia | Darwin | 3rd | 2021/8/16 | 2021/8/19 | 3 | Metropolitan area |
| Australia | Dubbo | 2nd | 2021/8/11 | 2021/10/11 | 61 | Local government area |
| Australia | Glen Innes Severn (NSW) | 2nd | 2021/8/14 | 2021/9/10 | 28 | Local government area |
| Australia | Glen Innes Severn (NSW) | 3rd | 2021/9/17 | 2021/9/24 | 7 | Local government area |
| Australia | Goulburn?Mulwaree | 2nd | 2021/8/14 | 2021/10/11 | 57 | Local government area |
| Australia | Greater Geelong | 1st | 2020/3/31 | 2020/5/12 | 43 | Local government area |
| Australia | Greater Geelong | 2nd | 2020/8/6 | 2020/9/16 | 41 | Local government area |
| Australia | Greater Geelong | 3rd | 2021/2/13 | 2021/2/17 | 5 | Local government area |
| Australia | Greater Geelong | 4th | 2021/5/28 | 2021/6/3 | 7 | Local government area |
| Australia | Greater Geelong | 5th | 2021/7/16 | 2021/7/27 | 12 | Local government area |
| Australia | Greater Geelong | 6th | 2021/8/5 | 2021/8/9 | 5 | Local government area |
| Australia | Greater Geelong | 7th | 2021/8/21 | 2021/9/9 | 20 | Local government area |
| Australia | Greater Geelong | 8th | 2021/9/20 | 2021/9/26 | 7 | Local government area |
| Australia | Greater Shepparton | 1st | 2020/3/31 | 2020/5/12 | 43 | Local government area |
| Australia | Greater Shepparton | 2nd | 2020/8/6 | 2020/9/16 | 41 | Local government area |
| Australia | Greater Shepparton | 3rd | 2021/2/13 | 2021/2/17 | 5 | Local government area |
| Australia | Greater Shepparton | 4th | 2021/5/28 | 2021/6/3 | 7 | Local government area |
| Australia | Greater Shepparton | 5th | 2021/7/16 | 2021/7/27 | 12 | Local government area |
| Australia | Greater Shepparton | 6th | 2021/8/5 | 2021/8/9 | 5 | Local government area |
| Australia | Greater Shepparton | 7th | 2021/8/21 | 2021/9/15 | 25 | Local government area |
| Australia | Greater Shepparton | 8th | 2021/10/2 | 2021/10/9 | 7 | Local government area |
| Australia | Greater Sydney | 2nd | 2021/6/26 | 2021/10/11 | 107 | Metropolitan area |
| Australia | Gunnedah | 2nd | 2021/8/14 | 2021/9/10 | 28 | Local government area |
| Australia | Gunnedah | 3rd | 2021/10/5 | 2021/10/11 | 6 | Local government area |
| Australia | Hilltops (NSW) | 2nd | 2021/8/14 | 2021/9/10 | 28 | Local government area |
| Australia | Hilltops (NSW) | 3rd | 2021/9/17 | 2021/10/1 | 14 | Local government area |
| Australia | Hunter Region | 2nd | 2021/8/5 | 2021/10/11 | 67 | Region |
| Australia | Illawarra | 2nd | 2021/6/26 | 2021/10/11 | 107 | Region |
| Australia | Latrobe Valley | 1st | 2020/3/31 | 2020/5/12 | 43 | Region |
| Australia | Latrobe Valley | 2nd | 2020/8/6 | 2020/9/16 | 41 | Region |
| Australia | Latrobe Valley | 3rd | 2021/2/13 | 2021/2/17 | 5 | Region |
| Australia | Latrobe Valley | 4th | 2021/5/28 | 2021/6/3 | 7 | Region |
| Australia | Latrobe Valley | 5th | 2021/7/16 | 2021/7/27 | 12 | Region |
| Australia | Latrobe Valley | 6th | 2021/8/5 | 2021/8/9 | 5 | Region |
| Australia | Latrobe Valley | 7th | 2021/8/21 | 2021/9/9 | 20 | Region |
| Australia | Latrobe Valley | 8th | 2021/9/29 | 2021/10/6 | 7 | Region |
| Australia | Lismore | 2nd | 2021/8/9 | 2021/9/10 | 32 | Local government area |
| Australia | Lismore | 3rd | 2021/9/16 | 2021/9/23 | 7 | Local government area |
| Australia | Lismore | 4th | 2021/10/3 | 2021/10/11 | 8 | Local government area |
| Australia | Melbourne | 1st | 2020/3/31 | 2020/5/12 | 43 | Metropolitan area |
| Australia | Melbourne | 2nd | 2020/7/9 | 2020/10/27 | 111 | Metropolitan area |
| Australia | Melbourne | 3rd | 2021/2/13 | 2021/2/17 | 5 | Metropolitan area |
| Australia | Melbourne | 4th | 2021/5/28 | 2021/6/10 | 14 | Metropolitan area |
| Australia | Melbourne | 5th | 2021/7/16 | 2021/7/27 | 12 | Metropolitan area |
| Australia | Melbourne | 6th | 2021/8/5 | 2021/10/21 | 78 | Metropolitan area |
| Australia | Mid-Coast | 2nd | 2021/8/14 | 2021/9/10 | 28 | Local government area |
| Australia | Mildura | 1st | 2020/3/31 | 2020/5/12 | 43 | Local government area |
| Australia | Mildura | 2nd | 2020/8/6 | 2020/9/16 | 41 | Local government area |
| Australia | Mildura | 3rd | 2021/2/13 | 2021/2/17 | 5 | Local government area |
| Australia | Mildura | 4th | 2021/5/28 | 2021/6/3 | 7 | Local government area |
| Australia | Mildura | 5th | 2021/7/16 | 2021/7/27 | 12 | Local government area |
| Australia | Mildura | 6th | 2021/8/5 | 2021/8/9 | 5 | Local government area |
| Australia | Mildura | 7th | 2021/8/21 | 2021/9/9 | 20 | Local government area |
| Australia | Mildura | 8th | 2021/10/9 | 2021/10/22 | 14 | Local government area |
| Australia | Mitchell Shire | 1st | 2020/3/31 | 2020/5/12 | 43 | Local government area |
| Australia | Mitchell Shire | 2nd | 2020/7/9 | 2020/9/16 | 70 | Local government area |
| Australia | Mitchell Shire | 3rd | 2021/2/13 | 2021/2/17 | 5 | Local government area |
| Australia | Mitchell Shire | 4th | 2021/5/28 | 2021/6/3 | 7 | Local government area |
| Australia | Mitchell Shire | 5th | 2021/7/16 | 2021/7/27 | 12 | Local government area |
| Australia | Mitchell Shire | 6th | 2021/8/5 | 2021/8/9 | 5 | Local government area |
| Australia | Mitchell Shire | 7th | 2021/8/21 | 2021/9/9 | 20 | Local government area |
| Australia | Mitchell Shire | 8th | 2021/9/20 | 2021/10/13 | 24 | Local government area |
| Australia | Muswellbrook | 2nd | 2021/8/5 | 2021/9/16 | 42 | Local government area |
| Australia | Muswellbrook | 3rd | 2021/9/28 | 2021/10/11 | 13 | Local government area |
| Australia | Northern Beaches (NSW) | 2nd | 2020/12/19 | 2021/1/10 | 22 | Local government area |
| Australia | Northern Beaches (NSW) | 3rd | 2021/6/26 | 2021/10/11 | 107 | Local government area |
| Australia | Oberon | 2nd | 2021/8/14 | 2021/9/10 | 28 | Local government area |
| Australia | Oberon | 3rd | 2021/9/29 | 2021/10/11 | 12 | Local government area |
| Australia | Peel | 2nd | 2021/1/31 | 2021/2/5 | 5 | Region |
| Australia | Peel | 3rd | 2021/4/23 | 2021/4/26 | 3 | Region |
| Australia | Peel | 4th | 2021/6/28 | 2021/7/2 | 4 | Region |
| Australia | Perth | 2nd | 2021/1/31 | 2021/2/5 | 5 | Metropolitan area |
| Australia | Perth | 3rd | 2021/4/23 | 2021/4/26 | 3 | Metropolitan area |
| Australia | Perth | 4th | 2021/6/28 | 2021/7/2 | 4 | Metropolitan area |
| Australia | Port Macquarie-Hastings | 2nd | 2021/8/14 | 2021/9/10 | 28 | Local government area |
| Australia | Port Macquarie-Hastings | 3rd | 2021/9/28 | 2021/10/5 | 7 | Local government area |
| Australia | Regional NSW | 2nd | 2021/8/14 | 2021/9/10 | 28 | Region |
| Australia | Rest of regional Victoria | 1st | 2020/3/31 | 2020/5/12 | 43 | State |
| Australia | Rest of regional Victoria | 2nd | 2020/8/6 | 2020/9/16 | 41 | State |
| Australia | Rest of regional Victoria | 3rd | 2021/2/13 | 2021/2/17 | 5 | State |
| Australia | Rest of regional Victoria | 4th | 2021/5/28 | 2021/6/3 | 7 | State |
| Australia | Rest of regional Victoria | 5th | 2021/7/16 | 2021/7/27 | 12 | State |
| Australia | Rest of regional Victoria | 6th | 2021/8/5 | 2021/8/9 | 5 | State |
| Australia | Rest of regional Victoria | 7th | 2021/8/21 | 2021/9/9 | 20 | State |
| Australia | Rest of the country | 1st | 2020/3/23 | 2020/5/15 | 52 | National |
| Australia | Richmond Valley | 2nd | 2021/8/9 | 2021/9/10 | 32 | Local government area |
| Australia | Snowy?Monaro | 2nd | 2021/8/14 | 2021/9/16 | 33 | Local government area |
| Australia | Snowy?Monaro | 3rd | 2021/9/30 | 2021/10/11 | 11 | Local government area |
| Australia | South Australia | 2nd | 2020/11/19 | 2020/11/22 | 3 | State |
| Australia | South Australia | 3rd | 2021/7/21 | 2021/7/27 | 7 | State |
| Australia | South Coast | 2nd | 2021/8/14 | 2021/10/11 | 57 | Region |
| Australia | South East Queensland | 2nd | 2021/6/29 | 2021/7/2 | 3 | Region |
| Australia | South East Queensland | 3rd | 2021/7/31 | 2021/8/8 | 8 | Region |
| Australia | South West | 2nd | 2021/1/31 | 2021/2/5 | 5 | Region |
| Australia | Southern Tasmania | 2nd | 2021/10/16 | 2021/10/19 | 3 | State |
| Australia | Surf Coast Shire | 1st | 2020/3/31 | 2020/5/12 | 43 | Local government area |
| Australia | Surf Coast Shire | 2nd | 2020/8/6 | 2020/9/16 | 41 | Local government area |
| Australia | Surf Coast Shire | 3rd | 2021/2/13 | 2021/2/17 | 5 | Local government area |
| Australia | Surf Coast Shire | 4th | 2021/5/28 | 2021/6/3 | 7 | Local government area |
| Australia | Surf Coast Shire | 5th | 2021/7/16 | 2021/7/27 | 12 | Local government area |
| Australia | Surf Coast Shire | 6th | 2021/8/5 | 2021/8/9 | 5 | Local government area |
| Australia | Surf Coast Shire | 7th | 2021/8/21 | 2021/9/9 | 20 | Local government area |
| Australia | Surf Coast Shire | 8th | 2021/9/20 | 2021/9/26 | 7 | Local government area |
| Australia | Tamworth | 2nd | 2021/8/9 | 2021/9/10 | 32 | Local government area |
| Australia | Townsville | 2nd | 2021/6/29 | 2021/7/2 | 3 | Metropolitan area |
| Australia | Townsville | 3rd | 2021/7/31 | 2021/8/8 | 8 | Metropolitan area |
| Australia | Upper Hunter | 2nd | 2021/8/5 | 2021/9/16 | 42 | Region |
| Australia | Yarrabah | 2nd | 2021/8/8 | 2021/8/11 | 3 | Local government area |
| Australia | Yass Valley | 2nd | 2021/8/14 | 2021/9/10 | 28 | Local government area |
| Australia | Yass Valley | 3rd | 2021/9/14 | 2021/9/27 | 14 | Local government area |
| Austria | Nationwide | 1st | 2020/3/16 | 2020/4/13 | 28 | National |
| Austria | Nationwide | 2nd | 2020/11/3 | 2020/11/30 | 27 | National |
| Austria | Nationwide | 3rd | 2020/12/26 | 2021/2/7 | 43 | National |
| Austria | Nationwide | 4th | 2021/11/15 | 2021/12/11 | 27 | National |
| Azerbaijan | Nationwide | 1st | 2020/3/31 | 2020/8/30 | 152 | National |
| Bangladesh | Nationwide | 1st | 2020/3/26 | 2020/5/16 | 51 | National |
| Bangladesh | Nationwide | 2nd | 2021/4/5 | 2021/7/14 | 100 | National |
| Bangladesh | Nationwide | 3rd | 2021/7/23 | 2021/8/10 | 18 | National |
| Barbados | Nationwide | 1st | 2020/3/28 | 2020/5/3 | 36 | National |
| Belgium | Nationwide | 1st | 2020/3/18 | 2020/5/4 | 47 | National |
| Belgium | Nationwide | 2nd | 2020/11/2 | 2020/12/14 | 42 | National |
| Belgium | Nationwide | 3rd | 2021/3/27 | 2021/4/26 | 30 | National |
| Bermuda | Nationwide | 1st | 2020/4/4 | 2020/5/2 | 28 | National |
| Bhutan | Nationwide | 1st | 2020/8/11 | 2020/9/1 | 21 | National |
| Bolivia | Nationwide | 1st | 2020/3/22 | 2020/7/31 | 131 | National |
| Botswana | Nationwide | 1st | 2020/4/2 | 2020/4/30 | 28 | National |
| Brazil | S?o Paulo | 1st | 2020/3/24 | 2020/5/10 | 47 | State |
| Brazil | Santa Catarina | 1st | 2020/3/17 | 2020/4/7 | 21 | State |
| Bulgaria | Nationwide | 1st | 2020/3/13 | 2020/6/15 | 94 | National |
| Bulgaria | Nationwide | 2nd | 2020/11/28 | 2021/1/31 | 65 | National |
| Bulgaria | Nationwide | 3rd | 2021/3/22 | 2021/3/31 | 10 | National |
| Cambodia | Phnom Penh | 1st | 2021/4/15 | 2021/5/5 | 21 | Municipality |
| Cambodia | Ta Khmau | 1st | 2021/4/15 | 2021/5/5 | 21 | Municipality |
| Canada | British Columbia | 1st | 2020/3/18 | 2020/5/18 | 61 | Province |
| Canada | British Columbia | 2nd | 2020/11/7 | 2021/1/8 | 62 | Province |
| Canada | Ontario | 1st | 2020/3/17 | 2020/5/14 | 58 | Province |
| Canada | Ontario | 3rd | 2021/4/3 | 2021/5/1 | 28 | Province |
| Canada | Ontario - North | 2nd | 2020/12/26 | 2021/1/9 | 14 | Region |
| Canada | Ontario - South | 2nd | 2020/12/26 | 2021/1/23 | 28 | Region |
| Canada | Ontario - South | 3rd | 2021/4/8 | 2021/6/2 | 55 | Region |
| Canada | Quebec | 2nd | 2020/12/25 | 2021/1/11 | 18 | Province |
| Colombia | Nationwide | 1st | 2020/3/25 | 2020/6/30 | 97 | National |
| Congo | Nationwide | 1st | 2020/3/31 | 2020/4/20 | 20 | National |
| Costa Rica | Nationwide | 1st | 2020/3/23 | 2020/5/1 | 39 | National |
| Croatia | Nationwide | 1st | 2020/3/18 | 2020/5/11 | 32 | National |
| Croatia | Nationwide | 2nd | 2020/12/22 | 2020/12/29 | 7 | National |
| Cyprus | Nationwide | 1st | 2020/3/24 | 2020/4/13 | 20 | National |
| Cyprus | Nationwide | 2nd | 2021/1/10 | 2021/1/31 | 21 | National |
| Cyprus | Nationwide | 3rd | 2021/4/26 | 2021/5/9 | 13 | National |
| Czech Republic | Nationwide | 1st | 2020/3/16 | 2020/4/12 | 27 | National |
| Czech Republic | Nationwide | 2nd | 2020/10/22 | 2021/3/28 | 174 | National |
| Denmark | Nationwide | 1st | 2020/3/12 | 2020/4/13 | 33 | National |
| Denmark | Nationwide | 2nd | 2020/12/25 | 2021/3/1 | 66 | National |
| Ecuador | Nationwide | 1st | 2020/3/16 | 2020/3/31 | 15 | National |
| El Salvador | Nationwide | 1st | 2020/3/12 | 2020/4/2 | 21 | National |
| Eritrea | Nationwide | 1st | 2020/4/2 | 2020/4/23 | 21 | National |
| Estonia | Nationwide | 1st | 2021/3/11 | 2021/4/11 | 31 | National |
| Fiji | Lautoka | 1st | 2020/3/20 | 2020/4/7 | 18 | City |
| Fiji | Lautoka | 2nd | 2021/4/19 |  |  | City |
| Fiji | Suva | 1st | 2020/4/3 | 2020/4/17 | 14 | City |
| Finland | Nationwide | 1st | 2021/3/8 | 2021/3/28 | 20 | National |
| France | Nationwide | 1st | 2020/3/17 | 2020/5/11 | 55 | National |
| France | Nationwide | 2nd | 2020/10/30 | 2020/12/15 | 46 | National |
| France | Nationwide | 3rd | 2021/4/4 | 2021/5/3 | 29 | National |
| France | Paris | 1st | 2020/3/17 | 2020/5/11 | 55 | Region |
| France | Paris | 2nd | 2020/10/30 | 2020/12/15 | 46 | Region |
| France | Paris | 3rd | 2021/3/19 | 2021/4/18 | 30 | Region |
| Georgia | Nationwide | 1st | 2020/3/31 | 2020/4/21 | 21 | National |
| Germany | Berchtesgadener Land | 1st | 2020/3/16 | 2020/5/30 | 76 | District |
| Germany | Berchtesgadener Land | 2nd | 2020/10/20 | 2020/11/3 | 14 | District |
| Germany | different regionally | 1st | 2020/3/16 | 2020/5/30 | 76 | National |
| Germany | different regionally | 2nd | 2020/11/2 | 2021/6/11 | 119 to 222 | National |
| Ghana | Accra | 1st | 2020/3/30 | 2020/4/12 | 13 | Metropolitan area |
| Ghana | Kumasi | 1st | 2020/3/30 | 2020/4/12 | 13 | Metropolitan area |
| Greece | Nationwide | 1st | 2020/3/23 | 2020/5/4 | 42 | National |
| Greece | Nationwide | 2nd | 2020/11/7 | 2021/3/22 | 135 | National |
| Greece | Serres | 1st | 2020/3/23 | 2020/5/4 | 42 | Regional unit |
| Greece | Serres | 2nd | 2020/11/3 | 2021/3/22 | 139 | Regional unit |
| Greece | Thessaloniki | 1st | 2020/3/23 | 2020/5/4 | 42 | Regional unit |
| Greece | Thessaloniki | 2nd | 2020/11/3 | 2021/3/22 | 139 | Regional unit |
| Guernsey | Nationwide | 1st | 2020/3/25 | 2020/6/20 | 87 | National |
| Guernsey | Nationwide | 2nd | 2021/1/23 | 2021/2/22 | 30 | National |
| Honduras | Nationwide | 1st | 2020/3/20 | 2020/5/17 | 58 | National |
| Hungary | Nationwide | 1st | 2020/3/28 | 2020/4/10 | 13 | National |
| India | Bengaluru | 2nd | 2021/4/27 | 2021/5/9 | 12 | Region |
| India | Delhi | 2nd | 2021/4/19 | 2021/5/31 | 42 | Region |
| India | Haryana | 2nd | 2021/5/3 | 2021/5/10 | 7 | State |
| India | Maharashtra | 2nd | 2021/4/14 | 2021/6/15 | 62 | State |
| India | Nationwide | 1st | 2020/3/25 | 2020/6/7 | 74 | National |
| India | Odisha | 2nd | 2021/5/5 | 2021/5/19 | 14 | State |
| India | Rajasthan | 2nd | 2021/5/10 | 2021/6/8 | 29 | State |
| India | Uttar Pradesh | 2nd | 2021/4/30 | 2021/5/10 | 10 | State |
| Iran | Nationwide | 1st | 2020/3/14 | 2020/4/20 | 37 | National |
| Iran | Nationwide | 2nd | 2021/4/14 | 2021/4/24 | 14 | National |
| Iraq | Nationwide | 1st | 2020/3/22 | 2020/4/11 | 20 | National |
| Ireland | All 26 counties | 1st | 2020/3/12 | 2020/5/18 | 67 | National |
| Ireland | All 26 counties | 2nd | 2020/10/21 | 2020/12/1 | 41 | National |
| Ireland | All 26 counties | 3rd | 2020/12/24 | 2021/4/12 | 119 | National |
| Ireland | Kildare | 1st | 2020/8/7 | 2020/8/31 | 24 | Regional |
| Ireland | Laois | 1st | 2020/8/7 | 2020/8/21 | 14 | Regional |
| Ireland | Offaly | 1st | 2020/8/7 | 2020/8/21 | 14 | Regional |
| Israel | Bnei Brak | 1st | 2020/4/2 | 2020/4/16 | 14 | City |
| Israel | Bnei Brak | 2nd | 2020/9/18 | 2020/10/18 | 30 | City |
| Israel | Bnei Brak | 3rd | 2020/12/27 | 2021/2/7 | 42 | City |
| Israel | Nationwide | 2nd | 2020/9/18 | 2020/10/18 | 30 | National |
| Israel | Nationwide | 3rd | 2020/12/27 | 2021/2/7 | 42 | National |
| Italy | Aosta Valley | 1st | 2020/3/9 | 2020/5/18 | 70 | Region |
| Italy | Aosta Valley | 2nd | 2020/11/6 | 2020/12/3 | 27 | Region |
| Italy | Aosta Valley | 3rd | 2021/1/17 | 2021/1/30 | 13 | Region |
| Italy | Calabria | 1st | 2020/3/9 | 2020/5/18 | 70 | Region |
| Italy | Calabria | 2nd | 2020/11/6 | 2020/12/3 | 27 | Region |
| Italy | Calabria | 3rd | 2021/1/17 | 2021/1/30 | 13 | Region |
| Italy | Lombardy | 1st | 2020/3/9 | 2020/5/18 | 70 | Region |
| Italy | Lombardy | 2nd | 2020/11/6 | 2020/12/3 | 27 | Region |
| Italy | Lombardy | 3rd | 2021/1/17 | 2021/1/30 | 13 | Region |
| Italy | Nationwide | 1st | 2020/3/9 | 2020/5/18 | 70 | National |
| Italy | Nationwide | 2nd | 2020/12/24 | 2021/1/6 | 13 | National |
| Italy | Nationwide | 3rd | 2021/3/15 | 2021/4/30 | 46 | National |
| Italy | Piedmont | 1st | 2020/3/9 | 2020/5/18 | 70 | Region |
| Italy | Piedmont | 2nd | 2020/11/6 | 2020/12/3 | 27 | Region |
| Italy | Piedmont | 3rd | 2021/1/17 | 2021/1/30 | 13 | Region |
| Italy | Province of Bolzano | 1st | 2020/3/9 | 2020/5/18 | 70 | Province |
| Italy | Province of Bolzano | 3rd | 2021/1/17 | 2021/1/30 | 13 | Province |
| Italy | Sicily | 1st | 2020/3/9 | 2020/5/18 | 70 | Region |
| Italy | Sicily | 3rd | 2021/1/17 | 2021/1/30 | 13 | Region |
| Jamaica | Saint Catherine | 1st | 2020/4/15 | 2020/4/22 | 7 | Parish |
| Jordan | Nationwide | 1st | 2020/3/18 | 2020/4/30 | 43 | National |
| Jordan | Nationwide | 2nd | 2020/11/10 | 2020/11/15 | 5 | National |
| Kosovo | Nationwide | 1st | 2020/3/14 | 2020/5/4 | 51 | National |
| Kuwait | Nationwide | 1st | 2020/5/10 | 2020/5/31 | 21 | National |
| Lebanon | Nationwide | 1st | 2020/3/15 | 2020/3/28 | 13 | National |
| Lebanon | Nationwide | 2nd | 2020/11/14 | 2020/11/28 | 14 | National |
| Libya | Nationwide | 1st | 2020/3/22 | 2020/6/27 | 97 | National |
| Lithuania | Nationwide | 1st | 2020/3/16 | 2020/6/18 | 94 | National |
| Lithuania | Nationwide | 2nd | 2020/11/7 | 2020/11/28 | 21 | National |
| Madagascar | Antananarivo | 1st | 2020/3/23 | 2020/4/20 | 28 | City |
| Madagascar | Toamasina | 1st | 2020/3/23 | 2020/4/20 | 28 | City |
| Malaysia | Nationwide | 1st | 2020/3/18 | 2020/6/9 | 83 | National |
| Malaysia | Nationwide | 2nd | 2021/1/13 | 2021/2/10 | 28 | National |
| Malaysia | Nationwide | 3rd | 2021/6/1 | 2021/8/16 | 76 | National |
| Mexico | Baja California | 1st | 2020/3/23 | 2020/6/1 | 70 | State |
| Mexico | Baja California | 2nd | 2020/12/7 |  |  | State |
| Mexico | Chihuahua | 1st | 2020/3/23 | 2020/6/1 | 70 | State |
| Mexico | Chihuahua | 2nd | 2020/10/23 | 2020/12/6 | 44 | State |
| Mexico | Durango | 1st | 2020/3/23 | 2020/6/1 | 70 | State |
| Mexico | Durango | 2nd | 2020/11/3 | 2020/12/6 | 33 | State |
| Mexico | Guanajuato | 1st | 2020/3/23 | 2020/6/1 | 70 | State |
| Mexico | Guanajuato | 2nd | 2021/1/4 |  |  | State |
| Mexico | Mexico City | 1st | 2020/3/23 | 2020/6/1 | 70 | State |
| Mexico | Mexico City | 2nd | 2020/12/19 |  |  | State |
| Mexico | Morelos | 1st | 2020/3/23 | 2020/6/1 | 70 | State |
| Mexico | Morelos | 2nd | 2021/1/4 |  |  | State |
| Mexico | Nationwide | 1st | 2020/3/23 | 2020/6/1 | 70 | National |
| Mexico | State of Mexico | 1st | 2020/3/23 | 2020/6/1 | 70 | State |
| Mexico | State of Mexico | 2nd | 2020/12/19 |  |  | State |
| Mongolia | Nationwide | 1st | 2020/3/10 | 2020/3/16 | 6 | National |
| Mongolia | Nationwide | 2nd | 2020/11/17 | 2020/12/1 | 15 | National |
| Montenegro | Tuzi | 1st | 2020/3/24 | 2020/5/5 | 42 | Municipality |
| Morocco | Nationwide | 1st | 2020/3/19 | 2020/6/10 | 83 | National |
| Myanmar | Yangon | 1st | 2020/4/18 | 2020/7/1 | 74 | City |
| Myanmar | Yangon | 2nd | 2020/9/1 | 2021/4/10 | 220 | City |
| Myanmar | Yangon | 3rd | 2021/7/8 | 2021/10/27 | 111 | City |
| Namibia | Nationwide | 1st | 2020/3/27 | 2020/5/4 | 38 | National |
| Nepal | Kathmandu | 1st | 2020/3/24 | 2020/7/21 | 120 | City |
| Nepal | Kathmandu | 2nd | 2020/8/20 | 2020/9/9 | 21 | City |
| Nepal | Nationwide | 1st | 2020/3/24 | 2020/7/21 | 120 | National |
| Netherlands | Nationwide | 1st | 2020/3/15 | 2020/4/6 | 22 | National |
| Netherlands | Nationwide | 2nd | 2020/12/15 | 2021/6/5 | 172 | National |
| Netherlands | Nationwide | 3rd | 2021/12/19 | 2022/1/14 | 26 | National |
| New Zealand | Auckland | 1st | 2020/3/23 | 2020/5/13 | 52 | Region |
| New Zealand | Auckland | 2nd | 2020/8/12 | 2020/8/30 | 19 | Region |
| New Zealand | Auckland | 3rd | 2021/2/14 | 2021/2/17 | 3 | Region |
| New Zealand | Auckland | 4th | 2021/2/28 | 2021/3/7 | 7 | Region |
| New Zealand | Auckland | 5th | 2021/8/18 | 2021/11/29 | 104 | Region |
| New Zealand | Nationwide | 1st | 2020/3/23 | 2020/5/13 | 52 | National |
| New Zealand | Nationwide | 2nd | 2021/8/18 | 2021/9/7 | 21 | National |
| Nigeria | Abuja | 1st | 2020/3/30 | 2020/4/12 | 13 | City |
| Nigeria | Lagos | 1st | 2020/3/30 | 2020/4/12 | 13 | City |
| Nigeria | Ogun | 1st | 2020/3/30 | 2020/4/12 | 13 | State |
| North Korea | Kaesong | 1st | 2020/7/25 | 2020/8/14 | 20 | City |
| Northern Cyprus | Nationwide | 1st | 2020/3/30 | 2020/5/4 | 35 | National |
| Oman | Jalan Bani Bu Ali | 1st | 2020/4/16 | 2020/5/29 | 43 | Province |
| Oman | Muscat | 1st | 2020/4/10 | 2020/5/29 | 49 | Governorate |
| Pakistan | Nationwide | 1st | 2020/3/24 | 2020/5/9 | 46 | National |
| Pakistan | Nationwide | 2nd | 2021/5/8 | 2021/5/18 | 10 | National |
| Panama | Nationwide | 1st | 2020/3/25 | 2020/5/31 | 67 | National |
| Papua New Guinea | Nationwide | 1st | 2020/3/24 | 2020/4/7 | 14 | National |
| Paraguay | Nationwide | 1st | 2020/3/20 | 2020/5/3 | 44 | National |
| Peru | Nationwide | 1st | 2020/3/16 | 2020/6/30 | 106 | National |
| Philippines | Cebu | 1st | 2020/3/27 | 2020/5/31 | 49 to 65 | Province |
| Philippines | Davao Region | 1st | 2020/3/19 | 2020/5/15 | 57 | Region |
| Philippines | Luzon | 1st | 2020/3/15 | 2020/5/31 | 46 to 61 to 77 | Island group |
| Philippines | Luzon | 2nd | 2020/8/4 | 2020/8/18 | 15 | Island group |
| Philippines | Soccsksargen | 1st | 2020/3/23 | 2020/5/15 | 53 | Region |
| Poland | Nationwide | 1st | 2020/3/13 | 2020/4/11 | 29 | National |
| Poland | Nationwide | 2nd | 2020/12/28 | 2021/1/17 | 20 | National |
| Poland | Nationwide | 3rd | 2021/3/20 | 2021/4/25 | 36 | National |
| Portugal | Nationwide | 1st | 2020/3/19 | 2020/4/2 | 14 | National |
| Portugal | Nationwide | 2nd | 2021/1/15 | 2021/3/15 | 59 | National |
| Qatar | Doha Industrial Area | 1st | 2020/3/11 | 2020/6/15 | 96 | Industrial park |
| Romania | Nationwide | 1st | 2020/3/25 | 2020/5/12 | 48 | National |
| Russia | Moscow | 1st | 2020/3/30 | 2020/5/12 | 43 | Metropolitan area |
| Russia | Moscow | 2nd | 2021/10/28 | 2021/11/4 | 7 | Metropolitan area |
| Russia | Rest of the country | 1st | 2020/3/28 | 2020/4/30 | 33 | National |
| Rwanda | Nationwide | 1st | 2020/3/21 | 2020/4/19 | 29 | National |
| Samoa | Nationwide | 1st | 2020/3/26 | 2020/4/8 | 13 | National |
| San Marino | Nationwide | 1st | 2020/3/14 | 2020/5/5 | 52 | National |
| Saudi Arabia | Jeddah | 1st | 2020/3/29 | 2020/6/21 | 84 | City |
| Saudi Arabia | Mecca | 1st | 2020/3/26 | 2020/6/21 | 87 | City |
| Saudi Arabia | Medina | 1st | 2020/3/26 | 2020/6/21 | 87 | City |
| Saudi Arabia | Qatif | 1st | 2020/3/9 | 2020/6/21 | 104 | Area |
| Saudi Arabia | Riyadh | 1st | 2020/3/26 | 2020/6/21 | 87 | City |
| Serbia | Nationwide | 1st | 2020/3/15 | 2020/5/4 | 37 to 50 | National |
| Singapore | Nationwide | 1st | 2020/4/7 | 2020/6/1 | 55 | National |
| Singapore | Nationwide | 2nd | 2021/5/16 | 2021/6/13 | 28 | National |
| Singapore | Nationwide | 3rd | 2021/7/22 | 2021/8/9 | 18 | National |
| Singapore | Nationwide | 4th | 2021/9/27 | 2021/10/24 | 28 | National |
| South Africa | Nationwide | 1st | 2020/3/26 | 2020/4/30 | 35 | National |
| South Africa | Nationwide | 2nd | 2020/12/28 | 2021/1/15 | 18 | National |
| South Africa | Nationwide | 3rd | 2021/6/28 | 2021/7/25 | 27 | National |
| Spain | Nationwide | 1st | 2020/3/14 | 2020/5/9 | 56 | National |
| Sri Lanka | Nationwide | 1st | 2020/3/18 | 2020/6/21 | 95 | National |
| Sri Lanka | Nationwide | 2nd | 2021/8/20 | 2021/10/1 | 42 | National |
| Switzerland | Nationwide | 1st | 2020/3/17 | 2020/4/27 | 41 | National |
| Switzerland | Nationwide | 2nd | 2021/1/18 | 2021/3/1 | 42 | National |
| Thailand | Nationwide | 1st | 2020/3/25 | 2020/5/31 | 67 | National |
| Trinidad and Tobago | Nationwide | 1st | 2020/3/17 | 2020/3/31 | 14 | National |
| Tunisia | Nationwide | 1st | 2020/3/22 | 2020/4/19 | 28 | National |
| Turkey | Cities | 1st | 2020/4/23 | 2020/4/27 | 4 | Only in 30 metropolitan cities and Zonguldak. |
| Turkey | Nationwide | 1st | 2021/4/29 | 2021/5/17 | 18 | National |
| Ukraine | Nationwide | 1st | 2020/3/17 | 2020/4/24 | 38 | National |
| United Arab Emirates | Nationwide | 1st | 2020/3/26 | 2020/4/17 | 22 | National |
| United Kingdom | Cambridgeshire | 1st | 2020/12/26 | 2021/3/29 | 93 | County |
| United Kingdom | East Ayrshire | 2nd | 2020/11/20 | 2020/12/11 | 21 | County |
| United Kingdom | East Dunbartonshire | 2nd | 2020/11/20 | 2020/12/11 | 21 | County |
| United Kingdom | East Midlands | 1st | 2020/12/31 | 2021/3/29 | 88 | Region |
| United Kingdom | East Renfrewshire | 2nd | 2020/11/20 | 2020/12/11 | 21 | County |
| United Kingdom | England | 1st | 2020/3/23 | 2020/7/4 | 103 | National |
| United Kingdom | England | 2nd | 2020/11/5 | 2020/12/2 | 27 | National |
| United Kingdom | England | 3rd | 2021/1/5 | 2021/3/28 | 83 | National |
| United Kingdom | Essex | 1st | 2020/12/26 | 2021/3/29 | 93 | County |
| United Kingdom | Glasgow | 2nd | 2020/11/20 | 2020/12/11 | 21 | County |
| United Kingdom | Kent & South East | 1st | 2020/12/20 | 2021/3/29 | 99 | Region |
| United Kingdom | Leicester | 1st | 2020/6/30 | 2020/7/24 | 24 | County |
| United Kingdom | London area | 1st | 2020/12/20 | 2021/3/29 | 99 | City |
| United Kingdom | Norfolk | 1st | 2020/12/26 | 2021/3/29 | 93 | County |
| United Kingdom | North East | 1st | 2020/12/31 | 2021/3/29 | 88 | Region |
| United Kingdom | North Lanarkshire | 2nd | 2020/11/20 | 2020/12/11 | 21 | County |
| United Kingdom | North West | 1st | 2020/12/31 | 2021/3/29 | 88 | Region |
| United Kingdom | Northern Ireland | 1st | 2020/3/23 | 2020/7/3 | 102 | Country |
| United Kingdom | Northern Ireland | 2nd | 2020/11/27 | 2020/12/11 | 14 | Country |
| United Kingdom | Northern Ireland | 3rd | 2020/12/26 | 2021/4/12 | 107 | Country |
| United Kingdom | Renfrewshire | 2nd | 2020/11/20 | 2020/12/11 | 21 | County |
| United Kingdom | Scotland | 1st | 2020/3/23 | 2020/6/29 | 98 | National |
| United Kingdom | Scotland | 2nd | 2020/12/26 | 2021/4/2 | 97 | National |
| United Kingdom | South Ayrshire | 2nd | 2020/11/20 | 2020/12/11 | 21 | County |
| United Kingdom | South Lanarkshire | 2nd | 2020/11/20 | 2020/12/11 | 21 | County |
| United Kingdom | Stirling | 2nd | 2020/11/20 | 2020/12/11 | 21 | County |
| United Kingdom | Suffolk | 1st | 2020/12/26 | 2021/3/29 | 93 | County |
| United Kingdom | Wales | 1st | 2020/3/23 | 2020/7/13 | 112 | Country |
| United Kingdom | Wales | 2nd | 2020/10/23 | 2020/11/9 | 17 | Country |
| United Kingdom | Wales | 3rd | 2020/12/20 | 2021/3/13 | 83 | Country |
| United Kingdom | West Dunbartonshire | 2nd | 2020/11/20 | 2020/12/11 | 21 | County |
| United Kingdom | West Lothian | 2nd | 2020/11/20 | 2020/12/11 | 21 | County |
| United Kingdom | West Midlands | 1st | 2020/12/31 | 2021/3/29 | 88 | Region |
| United States | California | 1st | 2020/3/19 | 2021/6/15 | 453 | State |
| United States | Connecticut | 1st | 2020/3/23 | 2020/4/22 | 30 | State |
| United States | Illinois | 1st | 2020/3/21 | 2020/5/30 | 70 | State |
| United States | Kansas City in Kansas | 1st | 2020/3/24 | 2020/4/19 | 26 | City |
| United States | Massachusetts | 1st | 2020/3/24 | 2020/5/4 | 41 | State |
| United States | Michigan | 1st | 2020/3/24 | 2020/4/13 | 20 | State |
| United States | New York | 1st | 2020/3/22 | 2020/6/13 | 83 | State |
| United States | Oregon | 1st | 2020/3/24 | 2020/5/15 | 53 | State |
| United States | Wisconsin | 1st | 2020/3/24 | 2020/5/13 | 50 | State |
| Venezuela | Nationwide | 1st | 2020/3/17 | 2020/5/13 | 57 | National |
| Vietnam | Bac Giang | 1st | 2020/4/1 | 2020/4/22 | 21 | 6 districts |
| Vietnam | Bac Giang | 2nd | 2021/5/18 |  | 23 | 6 districts |
| Vietnam | Bac Ninh | 1st | 2020/4/1 | 2020/4/22 | 21 | 4 districts and 1 city |
| Vietnam | Bac Ninh | 2nd | 2021/5/18 |  | 23 | 4 districts and 1 city |
| Vietnam | Da Nang | 1st | 2020/4/1 | 2020/4/22 | 21 | City |
| Vietnam | Da Nang | 2nd | 2020/7/28 | 2020/9/5 | 39 | City |
| Vietnam | Hai Duong | 1st | 2020/4/1 | 2020/4/22 | 21 | Province, Chi Linh city lockdown began from 28 January. |
| Vietnam | Hai Duong | 2nd | 2021/2/16 | 2021/3/2 | 14 | Province, Chi Linh city lockdown began from 28 January. |
| Vietnam | Nationwide | 1st | 2020/4/1 | 2020/4/22 | 21 | National |
| Zimbabwe | Nationwide | 1st | 2020/3/30 | 2020/5/2 | 33 | National |
