# Supplementary material for: Analyzing the worldwide progression of COVID-19 cases and deaths using nonlinear mixed-effects model
Source: PLoS One. 2024 Aug 12;19(8):e0306891. doi: 10.1371/journal.pone.0306891 (PMC11318863; doi:10.1371/journal.pone.0306891)
Supplement: S2 Table — (DOCX) [file pone.0306891.s007.docx]

**S2 Table. Lockdown start and end date in Japan in each place.** Downloaded from Wikipedia as of 15 Jan 2022, modified for clarity. (https://ja.wikipedia.org/wiki/%E7%B7%8A%E6%80%A5%E4%BA%8B%E6%85%8B%E5%AE%A3%E8%A8%80%E5%8F%8A%E3%81%B3%E3%81%BE%E3%82%93%E5%BB%B6%E9%98%B2%E6%AD%A2%E7%AD%89%E9%87%8D%E7%82%B9%E6%8E%AA%E7%BD%AE)[Japanese]

| **Country_territory** | **Place** | **Number of lockdown in the country** | **Lockdown Start date** | **Lockdown End date** | **Lockdown_Length (days)** | **Level** |
| --- | --- | --- | --- | --- | --- | --- |
| Japan | Nationwide | 1st | 2020/4/16 | 2020/5/14 | 28 | National |
| Japan | Chiba | 1st | 2020/4/7 | 2020/5/25 | 48 | City |
| Japan | Kanagawa | 1st | 2020/4/7 | 2020/5/25 | 48 | City |
| Japan | Saitama | 1st | 2020/4/7 | 2020/5/25 | 48 | City |
| Japan | Tokyo | 1st | 2020/4/7 | 2020/5/25 | 48 | City |
| Japan | Osaka | 1st | 2020/4/7 | 2020/5/21 | 44 | City |
| Japan | Hyogo | 1st | 2020/4/7 | 2020/5/21 | 44 | City |
| Japan | Fukuoka | 1st | 2020/4/7 | 2020/5/14 | 37 | City |
| Japan | Hokkaido | 1st | 2020/4/7 | 2020/5/25 | 48 | City |
| Japan | Kyoto | 1st | 2020/4/7 | 2020/5/21 | 44 | City |
| Japan | Tochigi | 1st | 2020/4/16 | 2020/5/14 | 28 | City |
| Japan | Aichi | 1st | 2020/4/16 | 2020/5/14 | 28 | City |
| Japan | Gifu | 1st | 2020/4/16 | 2020/5/14 | 28 | City |
| Japan | Okayama | 1st | 2020/4/16 | 2020/5/14 | 28 | City |
| Japan | Hiroshima | 1st | 2020/4/16 | 2020/5/14 | 28 | City |
| Japan | Okinawa | 1st | 2020/4/16 | 2020/5/14 | 28 | City |
| Japan | Ibaraki | 1st | 2020/4/16 | 2020/5/14 | 28 | City |
| Japan | Gunma | 1st | 2020/4/16 | 2020/5/14 | 28 | City |
| Japan | Shizuoka | 1st | 2020/4/16 | 2020/5/14 | 28 | City |
| Japan | Miyagi | 1st | 2020/4/16 | 2020/5/14 | 28 | City |
| Japan | Mie | 1st | 2020/4/16 | 2020/5/14 | 28 | City |
| Japan | Shiga | 1st | 2020/4/16 | 2020/5/14 | 28 | City |
| Japan | Chiba | 2nd | 2021/1/8 | 2021/3/21 | 72 | City |
| Japan | Kanagawa | 2nd | 2021/1/8 | 2021/3/21 | 72 | City |
| Japan | Saitama | 2nd | 2021/1/8 | 2021/3/21 | 72 | City |
| Japan | Tokyo | 2nd | 2021/1/8 | 2021/3/21 | 72 | City |
| Japan | Osaka | 2nd | 2021/1/14 | 2021/2/7 | 24 | City |
| Japan | Hyogo | 2nd | 2021/1/14 | 2021/2/7 | 24 | City |
| Japan | Fukuoka | 2nd | 2021/1/14 | 2021/2/7 | 24 | City |
| Japan | Hokkaido | 2nd | 2021/5/16 | 2021/6/20 | 35 | City |
| Japan | Kyoto | 2nd | 2021/1/14 | 2021/2/7 | 24 | City |
| Japan | Tochigi | 2nd | 2021/1/14 | 2021/2/7 | 24 | City |
| Japan | Aichi | 2nd | 2021/1/14 | 2021/2/28 | 45 | City |
| Japan | Gifu | 2nd | 2021/1/14 | 2021/2/28 | 45 | City |
| Japan | Okayama | 2nd | 2021/5/16 | 2021/6/20 | 35 | City |
| Japan | Hiroshima | 2nd | 2021/5/16 | 2021/6/20 | 35 | City |
| Japan | Okinawa | 2nd | 2021/5/23 | 2021/9/30 | 130 | City |
| Japan | Ibaraki | 2nd | 2021/8/20 | 2021/9/30 | 41 | City |
| Japan | Gunma | 2nd | 2021/8/20 | 2021/9/30 | 41 | City |
| Japan | Shizuoka | 2nd | 2021/8/20 | 2021/9/30 | 41 | City |
| Japan | Miyagi | 2nd | 2021/8/27 | 2021/9/12 | 16 | City |
| Japan | Mie | 2nd | 2021/8/27 | 2021/9/30 | 34 | City |
| Japan | Shiga | 2nd | 2021/8/27 | 2021/9/30 | 34 | City |
| Japan | Chiba | 3rd | 2021/8/2 | 2021/9/30 | 59 | City |
| Japan | Kanagawa | 3rd | 2021/8/2 | 2021/9/30 | 59 | City |
| Japan | Saitama | 3rd | 2021/8/2 | 2021/9/30 | 59 | City |
| Japan | Tokyo | 3rd | 2021/4/25 | 2021/6/20 | 56 | City |
| Japan | Osaka | 3rd | 2021/4/25 | 2021/6/20 | 56 | City |
| Japan | Hyogo | 3rd | 2021/4/25 | 2021/6/20 | 56 | City |
| Japan | Fukuoka | 3rd | 2021/5/12 | 2021/6/20 | 39 | City |
| Japan | Hokkaido | 3rd | 2021/8/27 | 2021/9/30 | 34 | City |
| Japan | Kyoto | 3rd | 2021/4/25 | 2021/6/20 | 56 | City |
| Japan | Tochigi | 3rd | 2021/8/20 | 2021/9/30 | 41 | City |
| Japan | Aichi | 3rd | 2021/5/12 | 2021/6/20 | 39 | City |
| Japan | Gifu | 3rd | 2021/8/27 | 2021/9/30 | 34 | City |
| Japan | Okayama | 3rd | 2021/8/27 | 2021/9/12 | 16 | City |
| Japan | Hiroshima | 3rd | 2021/8/27 | 2021/9/30 | 34 | City |
| Japan | Tokyo | 4th | 2021/7/12 | 2021/9/30 | 80 | City |
| Japan | Osaka | 4th | 2021/8/2 | 2021/9/30 | 59 | City |
| Japan | Hyogo | 4th | 2021/8/20 | 2021/9/30 | 41 | City |
| Japan | Fukuoka | 4th | 2021/8/20 | 2021/9/30 | 41 | City |
| Japan | Kyoto | 4th | 2021/8/20 | 2021/9/30 | 41 | City |
| Japan | Aichi | 4th | 2021/8/27 | 2021/9/30 | 34 | City |
