# Supplementary material for: Analyzing the worldwide progression of COVID-19 cases and deaths using nonlinear mixed-effects model
Source: PLoS One. 2024 Aug 12;19(8):e0306891. doi: 10.1371/journal.pone.0306891 (PMC11318863; doi:10.1371/journal.pone.0306891)
Supplement: S3 Table — (DOCX) [file pone.0306891.s008.docx]

**S3 Table. The tested factors list in the covariate modeling.** Downloaded from Our World in Data as of 15 Jan 2022 (https://covid.ourworldindata.org/data/owid-covid-data.csv) and from United Nations Statistics Division as of 31 March 2020 (https://unstats.un.org/UNSDWebsite).

| **Model parameter** | **Tested factors** |
| --- | --- |
| β | logarithmic of population, logarithmic of population density, logarithmic of capital city population (thousands), logarithmic of GDP per capita, logarithmic of extreme poverty, capital city population (as a percent of total population), urban population (percent), human development index, median age, proportion of aged 65 years or older, proportion of aged 70 years or older, life expectancy, capital latitude, average temperature |
| dr | logarithmic of population, logarithmic of population density, logarithmic of capital city population (thousands), logarithmic of GDP per capita, logarithmic of extreme poverty, capital city population (as a percent of total population), urban population (percent), human development index, median age, proportion of aged 65 years or older, proportion of aged 70 years or older, life expectancy, cardiovascular death rate, proportion of diabetes prevalence, proportion of female smokers, proportion of male smokers |
