# Supplementary material for: Analyzing the worldwide progression of COVID-19 cases and deaths using nonlinear mixed-effects model
Source: PLoS One. 2024 Aug 12;19(8):e0306891. doi: 10.1371/journal.pone.0306891 (PMC11318863; doi:10.1371/journal.pone.0306891)
Supplement: Attachment — Submitted filename: Response to Reviewes.docx [file pone.0306891.s009.docx]

May 8, 2024

Dr. Mohammad Nayeem Hasan

Editor-in-Chief
PLOS ONE

Dear Dr. Mohammad Nayeem Hasan,

Thank you for your instructions and forwarding the reviewer's thoughtful feedbacks regarding our manuscript, analyzing the worldwide progression of COVID-19 cases and deaths using nonlinear mixed-effects model. We agree with you that our paper needs some revision to meet PLOS ONE’s publication criteria, and we have amended this by changing the content. The amended contents are explained from next page. We also responded to all queries made by the reviewers and modified the manuscript appropriately.

With these changes to our final manuscript, we hereby resubmit our manuscript for a secondary evaluation. Thank you once again for your consideration of our paper.

Sincerely yours,

Hiroki Koshimichi, Ph.D.

Clinical Pharmacology & Pharmacokinetics,

Project Management Department,
Shionogi & Co., Ltd.,

8F, Nissay Yodoyabashi East Bldg., 3-13, Imabashi 3-chome,
Chuo-ku, Osaka, 541-0042, Japan

Phone: +81-70-7812-6705

e-mail: hiroki.koshimichi@shionogi.co.jp

Journal Requirements:

*1. Please ensure that your manuscript meets PLOS ONE's style requirements, including those for file naming. The PLOS ONE style templates can be found at*

*https://journals.plos.org/plosone/s/file?id=wjVg/PLOSOne_formatting_sample_main_body.pdf and*

*https://journals.plos.org/plosone/s/file?id=ba62/PLOSOne_formatting_sample_title_authors_affiliations.pdf*

>1

Thank you for your comment. I have followed PLOS ONE's style request and uploaded files with appropriate naming.

*2. Please note that PLOS ONE has specific guidelines on code sharing for submissions in which author-generated code underpins the findings in the manuscript. In these cases, all author-generated code must be made available without restrictions upon publication of the work. Please review our guidelines at https://journals.plos.org/plosone/s/materials-and-software-sharing#loc-sharing-code and ensure that your code is shared in a way that follows best practice and facilitates reproducibility and reuse.*

>2

I have uploaded model code file of the analysis to Github with open-source license. The link to the Github repository is added in manuscript Materials and Methods, Analysis section. (<https://github.com/h-koshi/WorldwideCovid19>)

*3. We note that Figure 7 in your submission contain map images which may be copyrighted. All PLOS content is published under the Creative Commons Attribution License (CC BY 4.0), which means that the manuscript, images, and Supporting Information files will be freely available online, and any third party is permitted to access, download, copy, distribute, and use these materials in any way, even commercially, with proper attribution. For these reasons, we cannot publish previously copyrighted maps or satellite images created using proprietary data, such as Google software (Google Maps, Street View, and Earth). For more information, see our copyright guidelines: http://journals.plos.org/plosone/s/licenses-and-copyright.*

*We require you to either (1) present written permission from the copyright holder to publish these figures specifically under the CC BY 4.0 license, or (2) remove the figures from your submission:*

*(1) You may seek permission from the original copyright holder of Figure 7 to publish the content specifically under the CC BY 4.0 license.*

*We recommend that you contact the original copyright holder with the Content Permission Form (http://journals.plos.org/plosone/s/file?id=7c09/content-permission-form.pdf) and the following text:*

*“I request permission for the open-access journal PLOS ONE to publish XXX under the Creative Commons Attribution License (CCAL) CC BY 4.0 (http://creativecommons.org/licenses/by/4.0/). Please be aware that this license allows unrestricted use and distribution, even commercially, by third parties. Please reply and provide explicit written permission to publish XXX under a CC BY license and complete the attached form.”*

*Please upload the completed Content Permission Form or other proof of granted permissions as an ""Other"" file with your submission.*

*In the figure caption of the copyrighted figure, please include the following text: “Reprinted from [ref] under a CC BY license, with permission from [name of publisher], original copyright [original copyright year].”*

*(2) If you are unable to obtain permission from the original copyright holder to publish these figures under the CC BY 4.0 license or if the copyright holder’s requirements are incompatible with the CC BY 4.0 license, please either i) remove the figure or ii) supply a replacement figure that complies with the CC BY 4.0 license. Please check copyright information on all replacement figures and update the figure caption with source information. If applicable, please specify in the figure caption text when a figure is similar but not identical to the original image and is therefore for illustrative purposes only.*

*The following resources for replacing copyrighted map figures may be helpful:*

*USGS National Map Viewer (public domain): http://viewer.nationalmap.gov/viewer/*

*The Gateway to Astronaut Photography of Earth (public domain): http://eol.jsc.nasa.gov/sseop/clickmap/*

*Maps at the CIA (public domain): https://www.cia.gov/library/publications/the-world-factbook/index.html and https://www.cia.gov/library/publications/cia-maps-publications/index.html*

*NASA Earth Observatory (public domain): http://earthobservatory.nasa.gov/*

*Landsat: http://landsat.visibleearth.nasa.gov/*

*USGS EROS (Earth Resources Observatory and Science (EROS) Center) (public domain): http://eros.usgs.gov/#*

*Natural Earth (public domain): http://www.naturalearthdata.com/*

>3

The map of Figure 7 was created with R package “maps v3.4.1”, in which the vector data of the world map was imported from public domain Natural Earth data project (the 1:50m world map, version 2.0, the latest version available in 2015).

I have revised Figure 7 to include the data source, as “Made with Natural Earth.” following the instruction https://www.naturalearthdata.com/about/terms-of-use/.

Reviewer #1

Dear Dr. Andrew Omame,

Thank you for providing your thoughtful insights. We have revised our manuscript based on your fruitful comments as below.

*1. Can authors provide more details on the specific nonlinear mixed-effect model based on the SIR framework that you employed?*

>1

In accord with the reviewer’s comment, I have uploaded the NONMEM model code of the analysis as supporting information to provide the detail of the model, which is now available on Github at <https://github.com/h-koshi/WorldwideCovid19>. I mentioned in the Method that the code is posted on Github.

*2. What led to the decision to limit the analysis to the period before the emergence of the Omicron variant, and how might this choice influence the generalizability of your findings?*

>2

Considering the reviewer's query, the below sentence was added in the Introduction section to clarify our objective in this study. (page 4, line 10)

"Due to the COVID-19, humanity experienced for the first time a disease with a rapid infection rate and high mortality rate, with limited effective treatments where information on disease spread is shared simultaneously around the world. Based on this premise, the purpose of this study is to precisely assess how various factors affect the spread of infection. The data after the emergence of the omicron variant was significantly less reliable for information on the number of infections due to the low severity of symptoms and mortality rates. It was excluded from this study because it violates this premise. “

The reasons for our choice to exclude the Omicron variant have also been explained in the discussion section, that is, lowered severity of the symptom and mortality rate, less reliable reported cases and deaths after the emergence of Omicron variant, and repeated infection cannot become negligible. Our study aimed to analyze infected cases and deaths with relatively high mortality rates by accounting for excess deaths, and thus the Omicron variant and its subtypes were considered unsuitable for inclusion in the analysis.

The below sentence was added in the materials and methods section for clarification. (page 5, line 29)

“The data after the emergence of Omicron variant was excluded from this analysis because of its lowered severity of the symptom and mortality rate, re-infection cannot be ruled out, but this model cannot be applied to re-infection cases.”

*3. Elaborate on how the assumption of changing effectiveness of lockdowns over time was incorporated into the model and its implications on the study outcomes.*

>3

Considering the reviewer's comment, the below sentences were added in the discussion section. (page 23, lines 6 and 15)

“In this study, effects by the various preventive measures of infection including lockdown were firstly evaluated as temporal inter-occasion variability. However, the estimated inter-occasion variability was high especially in the first year of the pandemic and gradually decreased to low levels, suggesting rapid changes in their effectiveness especially in the initial period. Therefore, we assumed that the lockdown effect should be estimated separately from the effect of other preventive measures. As a matter of fact, the lockdown effect was explained appropriately by this model and evaluated as negligible after approximately a year from the begging of the pandemic.”

“This study does not take into consideration the extent of lockdown measures, specifically the differences between national-level lockdowns and city-level lockdowns. Lockdowns are highly effective in preventing the spread of infections; however, they impose significant burdens on society, necessitating their implementation at the appropriate timing and level. Therefore, conducting a more detailed analysis of the duration and extent of lockdowns is considered useful in maximizing the efficiency of lockdown in future potential pandemic.”

*4. Could you explain in more detail how excess mortality was accounted for in your analysis and how it contributed to estimating unreported cases and deaths?*

>4

The method for accounting the excess mortality has been described in the Materials and Methods (Dataset), especially by Equation 1. Furthermore, I have uploaded the NONMEM model code of the analysis for more detailed understanding of how excess mortality was incorporated in this model and how it contributed to estimating unreported cases and deaths.

Significance of the excess death is described in the Discussion section as follows. (page 24, line 36)

“In this study, excess deaths enabled estimation of the actual mortality rate due to COVID-19. However, unlike confirmed cases and deaths (156 countries), excess death information was limited to 40 countries; therefore, the estimated model parameters for each country may be less reliable or biased for countries without excess death information than those with it. Furthermore, most of the countries whose excess deaths being available were Europe and developed countries, therefore, the bias in the distribution of excess death data in a particular country may lead to bias in the estimation of some model parameters. The predictive accuracy is expected to be improved by the inclusion of abundant and precise estimations of excess death data when infection is fatal and medical collapse occurs.”

It is difficult to state the extent to which the excess deaths affected the accuracy of the analysis, since there are large differences among countries, but it is possible that there was a significant impact in countries with serious infection situations, and that the accuracy of the analysis might be limited in some countries where there was no information on excess deaths.

*5. How were social responses, such as public compliance with preventive measures, integrated into the model, and how might these factors impact the validity of your results?*

>5

As the reviewer suggested, public compliance with preventive measures possibly affect the infection spread. However, it varies extensively country by country and thus it was very difficult to collect the information of detailed quantitative social responses from worldwide countries. Therefore, the social responses other than lockdown effect were modeled as random temporal inter-occasion variability in this model. Considering the reviewer's suggestion, the below sentence was added in the discussion section. (page 23, line 21)

“The social responses other than lockdown cannot be modeled in this study because it was difficult to collect the detailed quantitative information of social responses from worldwide countries, and they were assumed to be included in the random temporal inter-occasion variability.”

*6. Provide further insights into how GDP, proportion of people aged 65 or older, latitude, city population, and cardiovascular death rate were identified as significant influencing factors on mortality rates.*

>6

To respond the reviewer's query, the below sentence was modified in the discussion section. (page 22, line 21)

“Notably, they may include direct or indirect factors, and it is not possible to deny confounding between covariates. A careful interpretation is necessary.”

to

“Many economic, medical, population and geographic features of the nation are not independent index and correlated complexly with numerous characteristics. As a matter of fact, among the many correlated features, it is impossible and thus unimportant to identify which one has the particularly greatest impact on the spread of infection. Therefore, in this study, many country-specific characteristics were dimensionally compressed by the principal component analysis and analyzed impact of their independent component on the infection rate, and then a model was constructed to explain changes in the infection rate by limiting the analysis to the important factors among the detected components. As a result, we obtained interesting results showing that factors such as GDP and the proportion of aged people 65 years or older affect the infection rate. It should be noted, however, that it would be going too far to consider these factors as independent and distinct influencing factors considering the methodology of this study, and that they are representative of one of the similar characteristics of the trends and may have indirect effects and possible confounding.”

*7. Can you elaborate on how the odds ratios for transmissibility and mortality rates were derived for Beta, Gamma, and Delta variants, and how these results align with existing literature?*

>7

I have uploaded the NONMEM model code of the analysis to support understanding of how the odds ratios are estimated. Furthermore, the below sentence was added in the result and discussion section. (page 19, line 16, and page 24, line 5)

“with increased transmissibility of 29%, 25%, 38%, and 97% for Alpha, Beta, Gamma, and Delta variants, respectively”

“Furthermore, the estimated odds ratios may be different from the other reports since this study considers unreported cases and deaths in the calculation of odds ratios.”

*8. Clarify the methodology used to assess the impact of vaccination on mortality rates, and discuss any potential confounding variables that might influence these results.*

>8

I have uploaded the NONMEM model code of the analysis to clarify the methodology to assess the impact of vaccination on mortality rate. The below sentence was added in the discussion section. (page 24, line 24)

“This analysis of vaccine effectiveness is unique in that it integrates results from countries all over the world with very different characteristics, and quantitatively takes into account differences among countries in terms of lockdown, economic size, and the effects of viral mutations, which may have led to a more objective evaluation. On the other hand, the type and kind of vaccine could not be differentiated in this study because of the lack of data. Furthermore, the use of medication other than vaccine was not considered in this study, which may affect the estimation of the effect of vaccination.”

*9. Provide additional context on the relatively high odds ratio for mortality in the Delta variant compared to other variants, and discuss the potential implications for public health strategies.*

>9

The below sentence was added in the discussion section. (page 24, line 7)

“It is reported that the probability of hospitalization is high in Delta variant compared to the other variants [39], suggesting that the symptoms of COVID-19 are severe in patients infected with Delta variant. The difference of the estimated effect of vaccination on mortality rate between Delta variant and other variants may result from the difference in the severity of disease. The vaccination should be recommended as public health strategy especially when a high transmissibility or a high severity variant emerges.”

*10. Detail the sources of data used in your analysis, including information on the reliability and representativeness of the datasets for the countries considered.*

>10

Most of the data used in this study are from public source data, Our World in Data and Human Mortality Database, which has been collected very systematically and is generally based on the announcements of public institutions in each country. At present, there is no more reliable source of information regarding COVID-19. Some data including lockdown period in each country are sourced from Wikipedia. The data in Wikipedia are updated day by day, therefore, the lockdown period data used in this study are attached as supplementary data in S1 and S2 table.

*11. Can you conduct sensitivity analyses or validation studies to assess the robustness of your statistical methods, especially considering the complexity of the model?*

>11

Due to the large differences in characteristics among countries and the purpose of this study, which is to analyze all countries worldwide, we do believe that it is inappropriate to validate the model analysis using the data partitioning method. Instead, the parameter estimates and its confidence intervals were evaluated and provided in Table 1 and density plots are provided in S1 fig. Based on the results, we think the most parameter estimates in this study are reliable and the model is relatively robust.

Inspired by the reviewer's comment, we have performed sensitivity analysis on the effect of vaccination and obtained an interesting result. We have simulated the COVID-19 cases and deaths if there are no vaccine available in the world. The below sentence was added in the discussion section regarding this simulation. (page 24, line 30)

“For the purpose of testing the model parameter sensitivity, we have simulated the COVID-19 cases and deaths if there were no vaccines available in the world as supplementary data in S5 Fig. Based on the simulation, due to vaccination, the COVID-19 weekly cases and deaths would be decreased by approximately 70% by November 2021, about a year after the first vaccine became available. This suggests that early development of vaccines and their proactive administration are extremely important and had saved many lives.”

*12. Explicitly discuss the assumptions made in the model and their potential impact on the results, addressing any limitations or uncertainties associated with these assumptions.*

>12

This model have some assumptions, 1) all populations are susceptible, 2) transmissibility and mortality rate changes non-continuously considering various unascertained features of human society and butterfly effects, 3) lockdown effect decreases over time and its effect is the same if the lockdown level is at the national, state, or city level, 4) vaccination becomes effective immediately after the second dose of vaccination, and that the effect of vaccination declines gradually over time, 5) each country is relatively independent and has a similar informational significance, and 6) number of patients with repeated infection is negligible. We have already discussed the limitations or uncertainties in the manuscript except for 6. The below sentence was revised in the discussion section to clearly explain the model limitation. (page 25, line 20)

“This model does not consider the repeated infection, therefore, when examining patients with repeated infections, for example, the pandemic last for a long time enough to lose individual immunity against the virus or a variant emerges which have characteristics to escape the immunity, it is crucial to consider a model that accounts for the possibility of recovered individuals losing their immunity and rejoining susceptible populations.”

*13. Explore the temporal and regional variability in the effectiveness of infection prevention measures and discuss how these variations might influence your overall findings.*

>13

The below sentence was added in the discussion section. (page 23, line 31)

"In this study, the temporal and regional variability in the effectiveness of infection prevention measures were treated as a random inter-occasion variability, and their causal relationship is not captured. Fig 9 shows that during the first half year of the pandemic, large inter-occasion variability is estimated compared to the other periods, suggesting that there may have been significant differences in the temporal and regional variability in the effectiveness of infection prevention measures during this period.“

*14. Address potential biases in your study, such as reporting biases or selection biases, and discuss their implications for the interpretation of your results.*

>14

We think the lack of excess death data may lead to the bias of parameter estimates and misunderstanding of our results. The below sentence was modified in the discussion section for potential biases. (page 24, line 36)

“In this study, excess deaths enabled estimation of the actual mortality rate due to COVID-19. However, unlike confirmed cases and deaths (156 countries), excess death information was limited to 40 countries; therefore, the estimated model parameters for each country may be less reliable or biased for countries without excess death information than those with it. Furthermore, most of the countries whose excess deaths being available were Europe and developed countries, therefore, the bias in the distribution of excess death data in a particular country may lead to bias in the estimation of some model parameters.”

*15. In the conclusion, provide a more detailed discussion on the practical implications of your findings for policymakers and public health practitioners, including recommendations for future interventions.*

>15

The below sentence was added in the conclusion section. (page 25, line 33)

“This analysis showed that lockdown is a quite effective preventive measures of the transmission of infection in the beginning of the pandemic, however, the effect of lockdown decreases over time and reached to a negligible level after a certain period of time. The reason the lockdown quickly lost its effectiveness may be because society could not bear the strain, suggesting that combining various kind of more acceptable preventive measures are important, such as vaccination and medications. Therefore, development of vaccines and medicines are required as soon as possible after the spread of infectious disease in the world. Furthermore, to estimate the true mortality rate under the pandemic like COVID-19, to build a global system for demographic trend that allows us to understand excess deaths is also important.

*16. Check the suggested papers:* [*https://doi.org/10.1016/j.chaos.2020.110032*](https://doi.org/10.1016/j.chaos.2020.110032)*;* [*https://doi.org/10.1002/oca.2748*](https://doi.org/10.1002/oca.2748)*.*

>16

Thank you for sharing the useful papers. I have quoted the papers in this manuscript as references 16 and 17.

Reviewer #2:

Thank you for providing your thoughtful insights. We have revised our manuscript based on your fruitful comments as below.

*1. It is suggested to write a paragraph on death due to oxygen shortage in hospitals, in the introduction.*

>1

The below sentence was revised in the introduction section to state death due to the oxygen shortage in hospitals. (page 3, line 3)

“The mortality rate due to COVID-19 differs by period and country, and is influenced by the knowledge of COVID-19 infection, medical environment, as the oxygen shortage in hospitals might lead higher mortality rate at the beginning of pandemic [10], and patients background including age [11], viral strain, and vaccination.”

Reference 10:	Mansab F, Donnelly H, Kussner A, Neil J, Bhatti S, Goyal DK. Oxygen and Mortality in COVID-19 Pneumonia: A Comparative Analysis of Supplemental Oxygen Policies and Health Outcomes Across 26 Countries. Front Public Health. 2021 Jul 13:9:580585. doi: 10.3389/fpubh.2021.580585.

*2. Is there any source that provides the data of another health issue post-COVID-19?*

>2

The below sentence was added in the introduction section. (page 2, line 6)

“Now the COVID-19 is no longer a “global health emergency” [1], however, the virus is still circulating in the world and the risk remains of new variants emerging that cause new surges in cases and deaths, and other healthcare issues such as Long COVID remains [2]. Furthermore, information regarding infectious diseases other than COVID-19 and global health issues is being collected and made available [3].”

Reference 2:	Centers for Disease Control and Prevention [Internet]. Long COVID or Post-COVID Conditions. [Cited 31 Mar 2024]. Available from: https://www.cdc.gov/coronavirus/2019-ncov/long-term-effects/index.html

Reference 3:	World Health Organization [Internet]. WHO data collections. [Cited 31 Mar 2024]. Available from: https://www.who.int/data/collections
